# Tenofovir Disoproxil Fumarate for Prevention of HIV Infection in Women: A Phase 2, Double-Blind, Randomized, Placebo-Controlled Trial

**DOI:** 10.1371/journal.pctr.0020027

**Published:** 2007-05-25

**Authors:** Leigh Peterson, Doug Taylor, Ronald Roddy, Ghiorghis Belai, Pamela Phillips, Kavita Nanda, Robert Grant, Edith Essie Kekawo Clarke, Anderson Sama Doh, Renee Ridzon, Howard S Jaffe, Willard Cates

**Affiliations:** 1 Family Health International, Durham, North Carolina, United States of America; 2 Duke Clinical Research Institute, Durham, North Carolina, United States of America; 3 University of California San Francisco, San Francisco, California, United States of America; 4 J. David Gladstone Institutes, San Francisco, California, United States of America; 5 Ghana Health Service, Ministry of Health, Accra, Ghana; 6 University of Yaoundé, Yaoundé, Cameroon; 7 Bill and Melinda Gates Foundation, Seattle, Washington, United States of America; 8 Gilead Sciences, Foster City, California, United States of America

## Abstract

**Objectives:**

The objective of this trial was to investigate the safety and preliminary effectiveness of a daily dose of 300 mg of tenofovir disoproxil fumarate (TDF) versus placebo in preventing HIV infection in women.

**Design:**

This was a phase 2, randomized, double-blind, placebo-controlled trial.

**Setting:**

The study was conducted between June 2004 and March 2006 in Tema, Ghana; Douala, Cameroon; and Ibadan, Nigeria.

**Participants:**

We enrolled 936 HIV-negative women at high risk of HIV infection into this study.

**Intervention:**

Participants were randomized 1:1 to once daily use of 300 mg of TDF or placebo.

**Outcome measures:**

The primary safety endpoints were grade 2 or higher serum creatinine elevations (>2.0 mg/dl) for renal function, grade 3 or 4 aspartate aminotransferase or alanine aminotransferase elevations (>170 U/l) for hepatic function, and grade 3 or 4 phosphorus abnormalities (<1.5 mg/dl). The effectiveness endpoint was infection with HIV-1 or HIV-2.

**Results:**

Study participants contributed 428 person-years of laboratory testing to the primary safety analysis. No significant differences emerged between treatment groups in clinical or laboratory safety outcomes. Study participants contributed 476 person-years of HIV testing to the primary effectiveness analysis, during which time eight seroconversions occurred. Two were diagnosed in participants randomized to TDF (0.86 per 100 person-years) and six in participants receiving placebo (2.48 per 100 person-years), yielding a rate ratio of 0.35 (95% confidence interval = 0.03–1.93), which did not achieve statistical significance. Owing to premature closures of the Cameroon and Nigeria study sites, the planned person-years of follow-up and study power could not be achieved.

**Conclusion:**

Daily oral use of TDF in HIV-uninfected women was not associated with increased clinical or laboratory adverse events. Effectiveness could not be conclusively evaluated because of the small number of HIV infections observed during the study.

## Editorial Commentary

**Background:** The World Health Organization has estimated that in 2006 around 4.3 million people were newly infected with HIV. Infection rates seem to be increasing in some countries, and there is an urgent need to find safe and effective ways of preventing HIV from being transmitted from one person to another. Many strategies for the prevention of HIV transmission between adults, such as use of condoms or changes to behavior, are not completely reliable, and women, in particular, may not always be able to negotiate condom use. Additional strategies for reducing the risk of HIV transmission are needed. One of these strategies is called “pre-exposure prophylaxis.” This strategy involves individuals who are at high risk of becoming infected with HIV taking antiviral drugs to prevent HIV infection. One particular drug, tenofovir disoproxil fumarate, is currently approved as a treatment for HIV infection, and is also being investigated as a strong candidate for pre-exposure prophylaxis. The research presented here reports on results of a trial carried out at three different sites in Ghana, Cameroon, and Nigeria. In the trial, 936 women who were not infected with HIV but who were at high risk of becoming infected, were randomized to take tenofovir tablets daily or, alternatively, placebo tablets. The researchers planned to follow up with the women for 12 months, and the primary analysis for efficacy would focus on a comparison of the rate of new HIV infections between the two arms of the trial. Primary safety analyses included specific laboratory tests carried out on blood samples that might point to abnormalities in liver or kidney function. Safety data were also collected throughout the trial, and health problems that arose were classified as adverse events or serious adverse events.

**What this trial shows:** Unfortunately, this trial was not completed as planned. Two sites (Nigeria and Cameroon) were closed either before the planned number of participants had been recruited or before all participants had completed full follow-up. Therefore, not enough data were available from this trial to determine whether tenofovir reduced the risk of HIV infection. Only two sites contributed data for the primary safety analyses, which looked at liver and kidney function. The researchers did not see any statistically significant differences in these safety endpoints between participants taking tenofovir and those taking placebo. There were also no statistically significant differences between the treatment groups in the number of adverse events. The main efficacy analysis found two new HIV infections in the tenofovir group and six in the placebo group. Because only eight effectiveness endpoints were observed during this study, the difference in HIV incidence between these groups was not statistically significant.

**Strengths and limitations:** A strength of this trial is that it was correctly designed to address the original objectives of the study, involving appropriate concealment of randomization and blinding of participants and study staff to treatment assignment. The main limitation of this study was the closure of two study sites, which meant that the study did not have sufficient power to assess differences between trial arms in the primary efficacy analysis.

**Contribution to the evidence:** At the time this trial was completed, there was no other evidence from randomized studies that evaluated antiretroviral drugs for prevention of HIV infection. This trial cannot, however, definitively address whether tenofovir reduces the risk of HIV infection among at-risk women or not. Ongoing and future trials are essential in order to answer this question. The trial reported here provides important data on the safety of daily tenofovir among high-risk HIV-uninfected women; the safety data are encouraging and suggest that tenofovir use is not associated with increased adverse events as compared to placebo.

## INTRODUCTION

The HIV epidemic is continuing to grow worldwide [1]. Consistent and correct use of condoms is recommended for prevention of sexually transmitted HIV, but often women are unable to negotiate condom use with their male partners. Safe, effective, and easy to use methods of HIV prevention are urgently needed, especially for women.

Tenofovir disoproxil fumarate (TDF) [2], the orally bioavailable prodrug of tenofovir, is metabolized to a competitive inhibitor of viral reverse transcriptase. TDF was selected for clinical development as a treatment for HIV infection because of its (1) potency against wild-type HIV and some nucleoside-resistant strains of HIV [3–6], (2) low potential of selecting for TDF-resistant mutants [7], (3) low likelihood of metabolic/mitochondrial toxicity [8], and (4) pharmacologic profile supporting daily dosing [2]. TDF was licensed for the treatment of established HIV-l infection by the United States Federal Drug Administration in 2001, and the European Commission issued a marketing authorization in 2002 [9]. TDF has since been used worldwide for treatment of HIV infection, accounting for nearly 500,000 patient-years of observation [10].

We investigated the safety and effectiveness of a daily dose of 300 mg of TDF in preventing HIV infection among women at high risk for infection based on the following rationale: (1) initial prevention studies in simian models have provided support for both pre- and post-exposure efficacy of TDF in preventing retroviral infections [11–14]; (2) TDF has been shown to be safe in large numbers of HIV-infected persons [15,16]; (3) TDF is dosed conveniently once a day; (4) TDF has no known interactions with hormonal contraception [17]; (5) a high barrier to resistance was seen in clinical trials of HIV treatment, with the primary mutation identified (K65R) resulting in a reduction of viral replication to almost half that of wild-type [18]; and (6) the drug's sponsor, Gilead Sciences, is supportive of investigating the potential use of TDF as a preventive agent. Moreover, should it prove to be effective for HIV prevention, Gilead Sciences has committed to making TDF available in resource-poor settings for public health use, as they currently do for treatment of HIV, via no-profit pricing and licensing agreements.

## METHODS

### Participants

Study participants were recruited from areas within each city that were considered high HIV transmission areas, including markets, bars, and hotels. Although we did not specifically ask as part of the clinical trial procedures if the participants were sex workers, most exchanged sex for money. Special ethical considerations were taken into account because of the potential vulnerability of this population. We developed strategies to protect the confidentiality and autonomy of the participants, increase/ensure comprehension of the informed consent and research methods, and promote access to resources and services during and after the trial.

We enrolled HIV-antibody-negative women 18 to 35 y old who were at risk of HIV infection by virtue of having an average of three or more coital acts per week and four or more sexual partners per month. Participants had to be willing to use the study drug as directed and participate for up to 12 mo of follow-up. Because TDF has been associated with rare episodes of renal disorders, increased liver enzymes, and hypophosphatemia, participants were also required to have adequate renal function (serum creatinine < 1.5 mg/dl), liver function (aspartate aminotransferase [AST] and alanine aminotransferase [ALT] < 43 U/l), and serum phosphorus (≥2.2 mg/dl) at their screening visit. Since no adequate and well-controlled studies of TDF have been conducted in pregnant women, participants were not enrolled if pregnant or breastfeeding, or wishing to become pregnant during the 12 mo of study participation.

During recruitment, study staff explained the general purpose of the study and the eligibility requirements. If eligible, women were referred to the study clinic at each of the three study sites. At the screening visit, women completed written informed consent, received HIV pretest and condom counseling, and underwent oral mucosal transudate (OMT) rapid HIV testing. All participants received HIV post-test counseling, a physical and pelvic examination, urine pregnancy tests, and assessment of hepatic and renal function. Women with reactive OMT rapid HIV tests received ELISA to confirm HIV status. Potential participants were asked to return 4 wk after their screening visit to receive the results of their hepatic and renal function tests and, if applicable, a confirmatory HIV test. At this second visit, participants signed or marked a consent form for enrollment, received HIV counseling, provided urine for pregnancy testing, and provided another OMT sample for HIV testing.

All participants provided written informed consent in their preferred language. Illiterate participants were read the informed consent forms verbatim in the presence of a witness, and provided a mark or thumbprint in lieu of signature. The study protocol and informed consent forms were approved by the Ghana Health Service Ethical Review Committee, Accra, Ghana; the National Ethics Review Committee, Ministry of Public Health, Yaoundé, Cameroon; the University of Ibadan/University College Hospital Institutional Review Board, Ibadan, Nigeria; and the Protection of Human Subjects Committee, Family Health International (FHI), Durham, North Carolina, United States.

At each monthly follow-up visit, participants underwent OMT HIV and pregnancy testing, adverse event (AE) assessment, risk reduction counseling, and study drug and condom re-supply. Clinic staff counseled participants to take one pill every day, distributed condoms, and emphasized that condoms should be used for all sexual contacts with all partners because they were using a product of unknown or no effectiveness for preventing HIV. Participants responded to structured questionnaires on their sexual behavior and their experience taking the pills during the previous 30 d, and were reminded of study concepts discussed during the informed consent process. At months 1, 3, 6, 9, 12, and as needed, participants underwent physical examination and blood was drawn for laboratory assessment of hepatic and renal function. We discontinued product use for any participant who had a reactive rapid HIV or positive pregnancy test result. Pregnant women were allowed to resume study drug use after their pregnancy had ended, providing they were not breastfeeding. Study staff at each study site referred and offered to escort participants who became infected with HIV during the study to HIV-related psychological, social, and medical services, such as viral load, CD4 level, and HIV resistance testing in their communities, as well as antiretroviral drug provision when needed. Immediately after a participant missed a scheduled follow-up appointment, study staff made up to three attempts to contact that participant and reschedule the clinic appointment (ideally to occur within 1 wk of the original appointment).

### Objective and Interventions

The objective of this trial was to investigate the safety and preliminary effectiveness of a daily dose of 300 mg of TDF versus placebo in HIV-uninfected women.

### Outcomes

Protocol-defined primary safety endpoints included grade 2 or higher serum creatinine elevations (>2.0 mg/dl) for renal function, grade 3 or 4 AST or ALT elevations (>170 U/l) for hepatic function, and grade 3 or 4 phosphorus abnormalities (<1.5 mg/dl).

After the study began, the TDF prescribing information was amended to reflect a concern that people with chronic hepatitis B virus (HBV) infection are at risk of developing reactivation (i.e., flares) of hepatic disease after stopping use of TDF. To monitor for potential flares, we amended our protocol to add HBV surface antigen (HBsAg) testing for all enrolled participants at the time of product discontinuation, and ALT/AST monitoring for 3 mo after product discontinuation among HBsAg-positive participants. This amendment was implemented only at the Ghana site in August 2005, which was the only active study site (i.e., on drug) at the time.

The effectiveness endpoint was infection with HIV-1 or HIV-2, measured by detecting antibodies in OMT (OraQuick ADVANCE Rapid HIV-1/2 Antibody Test, Orasure Technologies, http://www.orasure.com) and confirmed by an enzyme-linked immunosorbent assay (ELISA) (Genetic Systems HIV-1/HIV-2 Plus O ELISA, Bio-Rad, http://www.bio-rad.com) or Western blot (Genetic Systems HIV-1 Western Blot, BioRad) from a finger prick or blood serum specimen. Discordant results between the rapid antibody and the ELISA assays were resolved with Western blot testing.

### Sample Size

We designed the study to have 90% power to conclude with 95% confidence that TDF reduced the rate of HIV infection by 50% (i.e., power to obtain a one-sided 95% upper confidence limit for the rate ratio that is less than 0.5) if the true rate of reduction due to TDF was at least 83%. The planned sample size was 1,200 participants (400 per site), with 12 mo of follow-up for each participant. Based on prior work with high-risk women in Cameroon [19], we assumed that the HIV incidence rate in the placebo group would be no less than five per 100 person-years, and that follow-up would be at least 80% at 12 mo. Under these assumptions, we expected that we would reach the required number of incident HIV infections (30) to achieve 90% power. Owing to premature closures of the Cameroon and Nigeria study sites, however, the planned person-years of follow-up and study power could not be achieved. Consequently, the primary effectiveness analysis was revised to a traditional two-sided test of non-zero effectiveness at the 0.05 significance level. The decision to change the primary effectiveness analysis was made by the project leader and statistician before unblinding the study.

For non-HIV safety outcomes, the planned sample size would have provided approximately 90% power to detect a doubling in the proportion of women experiencing any particular adverse reaction, if the proportion experiencing the reaction in the placebo arm was 0.05 or more. However, due to early termination of the study in Cameroon and Nigeria, we had only approximately 70% power to detect such a difference.

### Randomization and Blinding

A randomization manager not otherwise involved in the study developed a random allocation sequence using a permuted-block design stratified by site, with random block sizes of 12, 18, and 32. A copy of the randomization list was sent to the manufacturer, who filled each drug bottle with a 30-d supply of TDF or placebo (12 bottles per randomization number). Placebo tablets were identical to the TDF tablets in size, shape, color, and taste. Each drug bottle was marked with a sequential randomization number, but no product identifier. Participants, field study staff, monitors, statisticians, and other FHI staff involved in the trial were blinded to drug assignment. Study staff assigned each eligible participant the next sequential number, and gave her the first month's supply of study drug after she had fully qualified for the study and signed or marked the enrollment consent form.

### Statistical Methods

Primary safety analyses were based on time to grade 2 or higher abnormalities for creatinine or grade 3 or higher abnormalities for ALT, AST, and phosphorus, by site, using exact two-sided log-rank tests computed in StatXact Version 7 (Cytel, http://www.cytel.com). Because of irregularities in the performance of the laboratory in Nigeria, data from that site were excluded from the primary safety analyses. The project leader and study statistician made this decision before unblinding the study.

The primary effectiveness analysis was based on an exact log-rank test of no difference in the distribution of HIV-free survival times, with data pooled across all three sites. Each laboratory abnormality and HIV infection date was estimated as the midpoint between the date of the first positive test result and the previous, negative test date. A right censoring time of 12 mo was applied in Ghana, corresponding to the treatment regimen. For all participants in Cameroon and Nigeria, the censoring date was the date of permanent product withdrawal (4 February 2005 and 7 March 2005, respectively). Secondarily, exact confidence intervals for the relative risk of HIV, laboratory abnormalities, and AEs within system organ classes were computed under a Poisson assumption for the event rates in each treatment group. All primary and secondary analyses were based on two-sided tests and conducted at the 0.05 significance level.

Protocol-specified interim safety analyses occurred in April and December 2005. An independent Data Monitoring Committee, with access to treatment assignments, reviewed AEs and primary safety and effectiveness outcomes. The committee did not recommend changes in study procedures after either analysis.

FHI monitors (trained in Good Clinical Practice) visited the sites regularly to review study eligibility, informed consent, protocol compliance, laboratory procedures, source documents, and AEs. We attempted to get original hospital records, when available, for serious adverse events (SAEs) and deaths.

## RESULTS

### Participant Flow, Numbers Analyzed, and Baseline Characteristics

**Figure 1:**
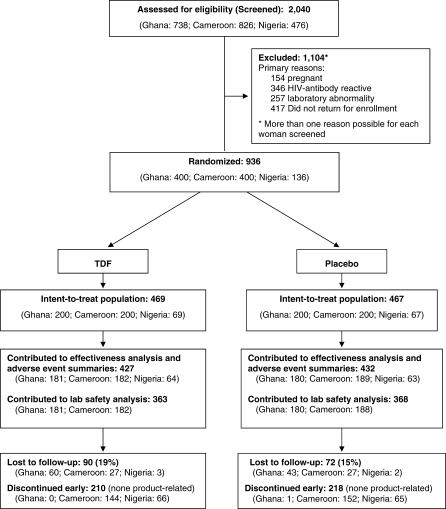
Participant Trial Flow Diagram

A total of 2,040 women were screened for inclusion in the study (Figure 1). The Ghana site completed planned enrollment (*n =* 400) in December 2004. Routine follow-up procedures continued monthly for 1 y, after which 3 mo of off-product monitoring of liver function was continued for the 56 HBsAg-positive participants. The Cameroon site completed enrollment (*n =* 400) in December 2004, before the Ministry of Health suspended study drug distribution in February 2005. In Nigeria, 136 of the planned 400 participants were enrolled before site closure. Loss to follow-up was 17.3% (19.2% in the TDF group and 15.4% in the placebo group, *p =* 0.11). Among these were 77 participants who never returned after their enrollment visit and were therefore excluded from the primary safety and effectiveness analyses. No participants were discontinued from the study by the site investigator or study clinician because of an AE or abnormal laboratory result. Participants enrolled into the TDF and placebo groups were similar in important demographic respects (Table 1).

**Table 1:**
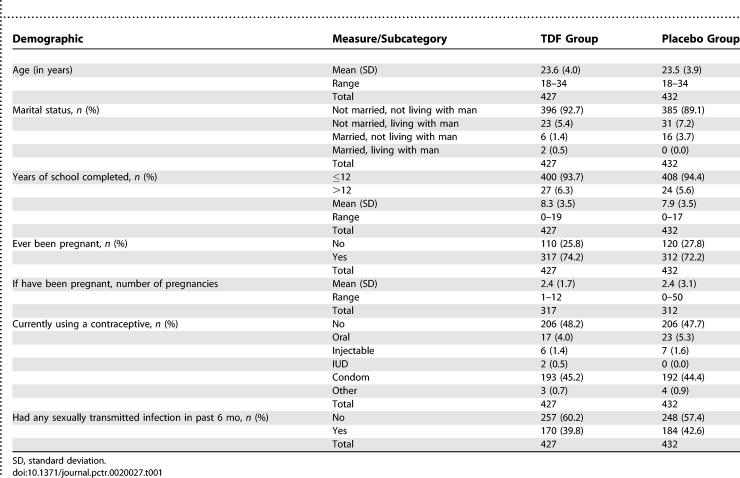
Demographic Characteristics

### Recruitment

We enrolled participants into this study between June 2004 and March 2005. The last participant visit occurred in Ghana in March 2006. In Cameroon, the Ministry of Health suspended study drug distribution in February 2005 primarily in response to concerns about the standard of long-term post-trial care that could be guaranteed to seroconverters. Participants continued to come to the clinic for off-product laboratory, pregnancy, and HIV testing, and HIV prevention counseling and condom distribution until September 2005, at which time the Cameroon site was closed. In Nigeria, because of repeated noncompliance with the protocol that was not resolved with staff retraining, enrollment was stopped in March 2005, and the site was closed thereafter.

### Sexual Behavior

During screening, participants reported an average of 12 coital acts per week with an average of 21 sexual partners in the previous 30 d (including 11 new partners). During follow-up, participants reported an average of 15 coital acts per week, with an average of 14 sexual partners in the previous 30 d (six new partners). Self-reported condom use increased from 52% at screening (average across all sites during the last coital act prior to screening) to approximately 92% at the enrollment, month 3, month 6, and month 9 visits, to 95% at the month 12 visit (for acts occurring during the last 7 d). The average condom use during the follow-up period was 92%. The overall pregnancy rate during follow-up was 52 per 100 person-years.

### Product Use

The amount of product used was estimated by subtracting the number of pills returned from the number dispensed, and dividing this number by the total number of days in the effectiveness analysis. Based on this calculation, drug was used for no more than 69% of all study days (78% in Cameroon, 68% in Ghana, and 50% in Nigeria). Excluding time off product due to pregnancy, drug was used for no more than 74% of study days. Missed/late clinic visits and pregnancy were the principal documented reasons for time off product.

### Safety

#### Hepatic and renal function.

Analysis of hepatic and renal function was restricted to data from Ghana and Cameroon, which resulted in 210.2 person-years of follow-up in the TDF group and 217.6 person-years in the placebo group. We did not find significant differences in the primary safety endpoints between treatment groups (Table 2). Specifically, none of the 363 participants assigned to TDF in Ghana or Cameroon had grade 3 or higher (>170 U/l) ALT or AST elevations before product withdrawal, whereas two and three of the 368 participants in the placebo group had grade 3+ ALT and AST elevations, respectively. The percentage of grade 1 or higher (>42 U/l) ALT and AST abnormalities was greater in the TDF group, but the difference did not achieve statistical significance. One participant in the TDF group had a grade 3+ decrease in phosphorus (<1.5 mg/dl), which spontaneously resolved to normal within 3 mo (study product was not withdrawn). No participants in either group had a grade 2+ creatinine elevation (>2.0 mg/dl). We did not find significant differences in laboratory abnormalities between treatment groups when the data were stratified by site, although significantly more grade 1 AST and phosphorus abnormalities occurred in Ghana than in Cameroon, and significantly more grade 1 creatinine abnormalities occurred in Cameroon than in Ghana.

**Table 2:**
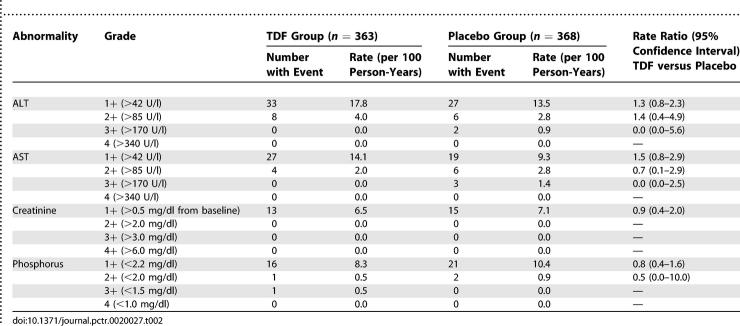
Laboratory Abnormalities during Scheduled Follow-Up in Ghana and Cameroon

Among the 56 participants who tested positive for HBsAg, 23 were in the TDF group and 33 in the placebo group. The mean and median ALT and AST levels were not significantly different between groups immediately before or after discontinuation of study drug. Four ALT/AST abnormalities (none over grade 1 [>42 U/l]) occurred within 3 mo after discontinuation of study drug in HBsAg-positive participants; one was in the TDF group and three were in the placebo group.

In Cameroon, study drug was stopped before the implementation of the protocol amendment that included HBsAg testing. We therefore monitored liver function for several months after product withdrawal in all participants, regardless of HBV status. The mean and median ALT and AST levels were not significantly different between groups immediately before or after discontinuation of study drug. Twenty ALT/AST abnormalities occurred within 3 mo after discontinuation of study drug; 14 were in the TDF group and six were in the placebo group. With the exception of one, all were grade 1 or 2 events (i.e., less than 85 U/l). One participant (who received TDF) had a grade 3 AST abnormality, which resolved within 1 mo.

#### Adverse events.

A total of 320 (75%) women in the TDF group and 310 (72%) women in the placebo group had at least one AE. The most frequently reported AEs (occurring in ≥5% of participants in either treatment group) are summarized in Table 3. There were no significant differences between treatment groups for any AEs within system organ classes. Similar results were obtained among HBsAg-positive participants.

**Table 3:**
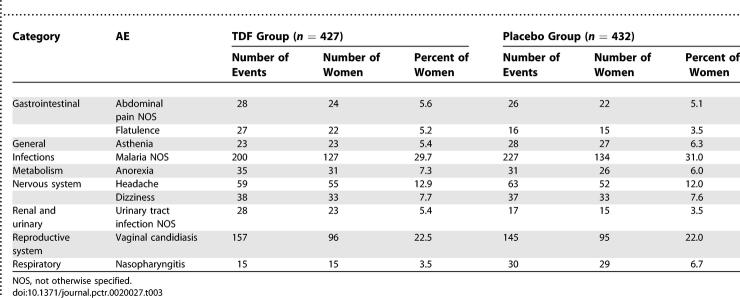
Selected Treatment-Emergent AEs Reported in ≥5% in Any Treatment Group (All Sites Combined)

Twenty–two SAEs were reported during the study (nine in the TDF group and 13 in the placebo group) in 17 participants. The majority of the SAEs were hospitalizations due to malaria (nine events). No SAEs were considered related to study drug. Two deaths occurred in Cameroon during the course of the study; both occurred approximately 5 mo after study drug dispensation was suspended. One was due to an unspecified condition with anemia (the participant had been randomized to placebo), and one was suspected to be related to an induced abortion (the participant had been randomized to TDF). Study drug was not discontinued in any participant by the site investigator or study clinician because of an AE or abnormal laboratory result.

### Effectiveness: HIV Incidence

For the primary effectiveness analysis, women in Cameroon, Ghana, and Nigeria contributed 232.6 person-years of follow-up in the TDF group and 241.3 person-years in the placebo group.

Eight seroconversions occurred among participants while receiving the study drug. Two HIV infections were diagnosed in participants randomized to TDF (rate = 0.86 per 100 person-years) and six in participants receiving placebo (rate = 2.48 per 100 person-years), yielding a rate ratio of 0.35 (95% confidence interval = 0.03–1.93; *p =* 0.24). With the exception of two participants (one randomized to TDF and one to placebo), all seroconversions were detected on or after the second monthly follow-up visit. Blood specimens were available from one of the two participants who seroconverted while on TDF; standard genotypic analysis revealed no evidence of drug resistance mutations. An additional six participants seroconverted after study drug was discontinued in Cameroon (four had been randomized to TDF and two to placebo).

## DISCUSSION

### Interpretation and Overall Evidence

Our data provide an encouraging rationale for additional research to evaluate oral antiretroviral drugs as prophylaxis against HIV infection. No significant differences in safety patterns occurred among participants receiving daily oral TDF compared with those receiving placebo, consistent with results seen in previous treatment studies [2].

No randomized clinical studies have been completed to evaluate the effectiveness of antiretroviral drugs as pre- or post-exposure prophylaxis against HIV infection. In a recent Cochrane review [20], only one case-control study of health-care workers after needlestick injury was identified. HIV-infected workers had significantly lower odds of having taken zidovudine prophylaxis after exposure than those who did not seroconvert (odds ratio = 0.19, 95% confidence interval = 0.06–0.52) [21]. The effectiveness of antiviral prophylaxis after sexual exposure is not known [22]. Non-randomized observations of post-exposure prophylaxis (PEP) are difficult to interpret because there could be underlying and uncontrolled differences between those who seek and use PEP and those who do not, including differences in behavior and access to information and medications. Furthermore, PEP or event-driven dosing is limited by the fact that failures can occur when treatment is not initiated because the risk of the exposure is not recognized [23]. We address this limitation of event-driven dosing in this study by recommending daily dosing for frequently exposed persons—a strategy referred to as pre-exposure prophylaxis. Because public health practice should be guided by evidence, especially in settings having competing demands for scarce public health resources, an urgent need exists for antiviral prophylaxis for HIV to be evaluated in randomized, placebo-controlled trials, as we report here. Further effectiveness studies in populations of women at high risk for acquiring HIV should proceed rapidly.

The premature stopping of the study in Cameroon and Nigeria limited the amount of follow-up safety and effectiveness data obtained in this study. Furthermore, AEs and laboratory abnormalities in the TDF group may have been diluted by lower than expected product use due to missed visits, drug stoppage due to pregnancy, and other reasons for non-use of study drug. The overall rate of HIV infection while women were on TDF or placebo in Ghana, Cameroon, and Nigeria was too low to demonstrate a reduction in risk for those assigned to the TDF group.

TDF is active against HBV, and is recommended for treatment of HBV infection in Europe and by many experts [24–27]. Transaminase increases have been observed in up to 25% of patients stopping anti-HBV drugs after receiving therapy for clinically important HBV infection, characterized by pre-treatment elevated ALT or AST or signs of liver fibrosis [28]. No flares of ALT or AST were observed among those with HBV infection in this study, although our analysis was limited to 23 TDF-treated women with reactive tests for HBsAg. The rate of HBV flares after withdrawing TDF may be low among people with normal baseline liver tests and no signs or symptoms of advanced liver disease, such as the women enrolled here. Additional data on the use of TDF in persons with circulating HBsAg are needed to confirm the results observed in this study.

We expected the HIV incidence in the placebo group to be no less than five per 100 person-years, over twice that we observed in this study. Thus, our power was less than anticipated. The lower than expected HIV incidence may be due in part to at least four factors: (1) the incidence rate was estimated from both our experience in earlier trials in a similar population and from current prevalence data; it was not specifically measured in each population before starting the study; (2) intense and consistent HIV/sexually transmitted infection prevention services and messages were provided to the study participants during the trial; (3) the effect of taking a pill every day for HIV prevention may have served as a timely reminder of imminent HIV risk such that participants modified their behavior to incorporate precautions against infection such as increased use of condoms; and (4) participants who elect to join clinical trials may be more inclined to safer behavior than those in their community who do not participate. Indeed, reductions of risk behavior have been observed in open label studies of PEP [23,29].

### Generalizability

As a new HIV prevention approach, prophylactic use of TDF could be used with other prevention strategies such as condoms to reduce the number of people who become infected with HIV. Larger phase 3 studies to conclusively determine the safety, effectiveness, and feasibility of using TDF (either alone or in combination with other antiretrovirals) as chemoprophylaxis against HIV infection in both women and men are needed.

## SUPPORTING INFORMATION

CONSORT Checklist Click here for additional data file. (49 KB DOC)

Original Trial Protocol Click here for additional data file. (269 KB PDF)

Amended Trial Protocol Click here for additional data file. (364 KB PDF)

## Abbreviations

AE: adverse event
ALT: alanine aminotransferase
AST: aspartate aminotransferase
ELISA: enzyme-linked immunosorbent assay
FHI: Family Health International
HBsAg: hepatitis B virus surface antigen
HBV: hepatitis B virus
OMT: oral mucosal transudate
PEP: post-exposure prophylaxis
SAE: serious adverse event
TDF: tenofovir disoproxil fumarate
