# Supplementary material for: Tenofovir Disoproxil Fumarate for Prevention of HIV Infection in Women: A Phase 2, Double-Blind, Randomized, Placebo-Controlled Trial
Source: PLoS Clin Trials. 2007 May 25;2(5):e27. doi: 10.1371/journal.pctr.0020027 (PMC1876601; doi:10.1371/journal.pctr.0020027)
Supplement: Original Trial Protocol [file pctr.0020027.sd002.pdf]

Phase 2 Study of Tenofovir Disoproxil Fumarate (TDF) for Prevention of HIV

Study 9780

**Funded by:**	Bill and Melinda Gates Foundation
**Sponsor:**	Family Health International<br>PO Box 13590<br>Research Triangle Park, NC 27709<br>Tel. 1.919.544.7040
**Pharmaceutical Sponsor:**	Gilead Sciences, Inc.<br>333 Lakeside Drive<br>Foster City, CA 94404
**Investigational Product:**	Tenofovir Disoproxil Fumarate (TDF)
**FHI Project Leader:**	Ronald E. Roddy<br>Senior Epidemiologist
**FHI Study Clinician:**	Dr. Kavita Nanda<br>Associate Medical Director<br>PO Box 13590<br>Research Triangle Park NC 27709<br>Tel: 1.919.544.7040
**Investigators:**	Dr. Anderson Sama Doh (Coordinating Investigator, Cameroon)<br>Professor of Obstetrics and Gynaecology<br>Faculty of Medicine and Biomedical Sciences<br>University of Yaoundé 1<br>(237) 2 220-14-59;(237)770-11-24<br>(237)221-24-38 ; (237)231-73-44<br>Fax : (237)221-24-30

Dr. Ekani Jean Marie Edengue
Chief Medical Officer
District de Sante de New Bell
CMA Congo II
Douala, Cameroun
Tel: (237)-342 17 66/(237) 998-03-48

Dr. Ayodele Olatunji Arowojolu
Department of Obstetrics and Gynaecology
University College Hospital
Ibadan, Nigeria

Dr. Edith Essie Kekawo Clarke
Head of Occupational Health Unit
Ministry of Health, Ghana

Clinical Laboratories:

Cameroon
Centre Pasteur du Cameroun
BP 1274
Yaoundé, Cameroon

Nigeria
Obstetrics and Gynaecology Laboratory
University College Hospital
Ibadan, Nigeria

Ghana
Tema General Hospital
Hospital Street
Tema

And

Virology Unit
Noguchi Memorial Institute for Medical Research
University of Ghana, Legon

Phase 2 Study of Tenofovir Disoproxil Fumarate (TDF) for Prevention of HIV

Study 9780

I, the Investigator, agree to conduct this study in full accordance with the provisions of this protocol and will comply with all requirements regarding the obligations of clinical investigators as fully outlined in the Declaration of Helsinki and in the Statement of Investigator, which I have also signed. I agree to maintain all study documentation until Family Health International (FHI) advises that it is no longer necessary. I also agree to publish or present data only upon review and after discussion with FHI.

I have read and understand the information in this protocol, including the potential risks and side effects of the product under investigation, and will ensure that all associates, colleagues, and employees assisting in the conduct of the study are informed about the obligations incurred by their contribution to the study

________________________________ ________________________________

Signature of Investigator Date

Prepared by:

Clinical Research Division
Family Health International
P.O. Box 13950
Research Triangle Park, NC 27709 USA

2224 E. NC Highway 54
Durham, NC 27713 USA

Tel: 1.919.544.7040
FAX: 1.919.544.7261

TABLE OF CONTENTS

STUDY SUMMARY 7
LIST OF ABBREVIATIONS AND ACRONYMS 8
1. INTRODUCTION 9
1.1 Background 9
1.2 Rational 10
2. STUDY OBJECTIVES 11
2.1 Primary Objectives 11
3. TRIAL DESIGN 11
3.1 Primary Endpoints 11
3.2 Study Design 11
3.3 Measures to Minimize Bias 12
3.3.1 Randomization 12
3.3.2 Allocation concealment 12
3.3.3 Masking 12
3.3.4 Decoding procedure 12
3.4 Study Product 12
3.5 Duration of the Study 14
3.6 Withdrawal Criteria 14
3.6.1 Individual participants 14
3.6.2 Study phases 15
3.6.3 Study 15
3.7 Study Product Accountability 15
3.8 Randomization Codes 16
3.9 Data Directly Recorded on DCFs 16
4.0 SELECTION OF PARTICIPANTS 16
4.1 Eligibility Criteria 16
4.2 Loss to Follow-up Procedures 17
5.0 STUDY PRODUCTS AND TREATMENTS 17
5.1 Products 17
5.2 Concomitant Treatments 18
5.3 Monitoring of Product Compliance 18
6.0 EFFECTIVENESS AND SAFETY ASSESSMENT 19
6.1 Effectiveness Parameters 19
6.2 Methods and Timing of Effectiveness Parameters 19
6.3 Safety Measurements 19
6.4 Methods and Timing of Safety Measurements 20
7.0 ADVERSE EVENTS AND REPORTING REQUIREMENTS 20
7.1 Adverse Events 20
7.2 Relationship of adverse event to study product 21
7.3 Reporting adverse events 22
7.4 Serious Adverse Events 22
7.5 Reporting Serious Adverse Events 23
7.6 Adverse Event Management 24

7.6.1 Grades 1 and 2 laboratory abnormality or clinical event (except serum creatinine elevation) ... 24
7.6.2 Grade 3 laboratory abnormality or clinical event (except serum creatinine elevation) ... 24
7.6.3 Grade 4 laboratory abnormality or clinical event (except serum creatinine elevation) ... 24
7.6.4 Grade 1 serum creatinine elevation ... 25
7.6.5 Grades 2-4 serum creatinine elevations ... 25
7.7 Social Risk Events ... 25
8.0 STUDY VISIT SUMMARY ... 25
8.1 Screening Visit ... 25
8.2 Enrollment Visit ... 26
8.3 Follow-up Visits ... 27
8.4 Interim Contacts and Unscheduled Visits ... 27
9.0 STATISTICAL SUMMARY ... 28
9.1 Study Size Justification ... 28
9.1.1 Effectiveness outcome ... 28
9.1.2 Safety outcomes ... 28
9.2 Analysis Plan Summary ... 29
9.2.1 Analysis of effectiveness outcomes ... 29
9.2.2 Safety outcomes ... 29
9.3 Interim Analysis Plan Summary ... 30
10.0 MONITORING PLAN SUMMARIES ... 30
10.1 Clinical Monitoring Plan ... 30
10.2 Safety Monitoring Plan ... 31
10.3 Data Monitoring Committee ... 31
11.0 DATA MANAGEMENT PLAN SUMMARY ... 31
12.0 PROTOCOL VIOLATIONS ... 32
12.1 Protocol Violations ... 32
13.0 STUDY DOCUMENTS ... 32
13.1 Study Initiation ... 32
13.2 Study Conduct ... 33
13.3 Study Completion ... 34
13.4 Site Record Retention and Access to Documents at the Site ... 34
14.0 QUALITY CONTROL AND QUALITY ASSURANCE ... 34
14.1 Quality Control of the OMT Sampling ... 34
14.2 Quality Assurance ... 35
15.0 ETHICS AND RESEARCH INTEGRITY ... 35
15.1 Institutional Review Board Review and Approval ... 35
15.2 Informed Consent ... 35
15.3 Participant Confidentiality ... 36
15.4 Research Integrity ... 36
16.0 PUBLICATION POLICY ... 36
17.0 ADDITIONAL INVESTIGATOR RESPONSIBILITIES ... 37
18.0 REFERENCES ... 37
APPENDIX 1: COMMON TOXICITY GRADING SCALE ... 39

APPENDIX 2: MANAGEMENT OF CLINICAL AND LAB ADVERSE EVENTS................. 49
APPENDIX 3: MANAGEMENT OF CREATININE ELEVATIONS.. 50
APPENDIX 4: STUDY ACTIVITIES CHART... 51

STUDY SUMMARY

Phase 2 Study of Tenofovir Disoproxil Fumarate (TDF) for Prevention of HIV

Study 9780

**Design:**	Phase 2, multi-center, fully-masked, randomized, parallel, placebo-controlled effectiveness and extended safety study to assess the role of TDF as prophylaxis to prevent HIV acquisition
**Population:**	1,200 HIV antibody negative participants:<br>600 participants will use TDF daily<br>600 participants will use placebo daily
**Study Duration:**	6 months screening, 6 months recruitment, 12 months of product use for each participant, 20 months in the field, including screening and close-down
**Primary Objectives:**	Determine the effectiveness and safety of daily use of 300 mg TDF for HIV prevention
**Primary Endpoints:**	Incidence of HIV-1 and HIV-2 infection as determined by detection of HIV antibodies from oral mucosal transudate (OMT) specimens and confirmation by ELISA.<br><br>Incidence of liver and kidney events as determined by AST and ALT for hepatic function, and serum creatinine and phosphorus for kidney function.
**Study Sites:**	Douala, Cameroon<br>Ibadan, Nigeria<br>Tema, Ghana

LIST OF ABBREVIATIONS AND ACRONYMS

AE	adverse event
AIDS	acquired immunodeficiency syndrome
ALT (SGPT)	alanine aminotransferase
AST (SGOT)	aspartate aminotransferase
DCF	data collection forms
DMC	Data Monitoring Committee
eDCF	electronic data collection form
ELISA	enzyme-linked immunosorbent assay
FDA	(U.S.) Food and Drug Administration
g	gram(s)
GCP	Good Clinical Practice guidelines
HAART	highly active antiretroviral therapy
HIV-1	human immunodeficiency virus type 1
HIV-2	human immunodeficiency virus type 2
ICH	International Conference of Harmonisation
IRB	Institutional Review Board
ITT	intent-to-treat
IU	international units
kg	kilogram(s)
LLN	lower limit of the normal range
LLOQ	lower limit of quantification
mg	milligram(s)
mL	milliliter(s)
mm3	cubic millimeter(s)
NNRTI	non-nucleoside reverse transcriptase inhibitor
NRTI	nucleoside reverse transcriptase inhibitor
NtRTI	nucleotide reverse transcriptase inhibitor
OMT	oral mucosal transudate
PCR	polymerase chain reaction
PharmaLinkFHI	a contract research organization responsible for data management
PrEP	pre-exposure prophylaxis
RT	reverse transcriptase
SAE	serious adverse event
TDF	tenofovir disoproxil fumarate, GS-4331-05, PMPA prodrug
µg	microgram
ULOQ	upper limit of quantification
ULN	upper limit of the normal range

1. INTRODUCTION

1.1 Background

The HIV epidemic is continuing to grow worldwide. An effective, easy to use method of prevention is urgently needed. Consistent and correct condom use is the only method currently recommended for prevention of sexually transmitted HIV. Vaginal microbicides are being developed in the hopes of offering women a method to protect themselves from infection when condom use is not possible. However, no vaginal microbicide candidate has shown effective protection from HIV in human trials.

TDF is an orally bioavailable prodrug of tenofovir, an acyclic nucleotide analog with activity in vitro against retroviruses, including HIV-1, HIV-2, and hepadnaviruses.[1]

The non-clinical toxicology of TDF has been studied in mice, rats, rabbits, and dogs. The principal target organs of toxicity following oral administration of TDF were the gastrointestinal tract, kidney, and bone.[2]

The safety profile of TDF is primarily derived from clinical studies. There has been no evidence of renal toxicity related to TDF. Laboratory evaluation of serum creatinine and phosphorus has not demonstrated a difference between TDF and placebo or other active controls.[2]

Evidence suggests that oral, once-daily TDF 300 mg appears comparable to placebo for safety and has durable efficacy in HIV-1-infected patients with high levels of baseline resistance to nucleoside reverse transcriptase inhibitors. The FDA has approved 300 mg TDF per day as safe and effective for treatment for HIV infection. TDF has received marketing approval in the European Union. No marketing authorizations have been withdrawn or suspended, and no marketing authorizations have been denied. No clinical trials have been suspended, there have been no dosage modifications since approval, and no changes in target populations, labeling, or formulation changes for safety reasons since approval.[2]

Furthermore, animal studies provide strong support for both the pre-exposure as well as post-exposure efficacy of TDF in preventing retroviral infections. These studies include challenge by both the intravenous and intravaginal modes of SIV infection as well as vertical transmission to neonate macaques.

Tenofovir was found to have potent activity in an acute challenge experiment of simian immunodeficiency virus (SIV) infection in monkeys, a model of needlestick injury. When treatment was started as late as 24 hours after intravenous SIV inoculation and continued for four weeks, tenofovir was found to completely protect the animals from SIV infection without signs of toxicity.[3]

By intravaginal administration topically, tenofovir also was completely protective against vaginal transmission of SIV in another simian model. Such activity of an antiviral compound in

animal models is unprecedented and makes tenofovir a promising agent for both treatment and prevention of HIV infection.[4]

The safety of TDF has been established with regard to its use in combination with other antiretrovirals for HIV-infected persons. However, the safety profile for healthy, uninfected persons or for uninfected persons with conditions such as malaria has not been established.

This Phase 2 study will evaluate the effectiveness of TDF 300 mg as an HIV prevention method when taken once a day. This Phase 2 study in three countries will provide extended safety data for high-risk populations in those countries. The daily tablet of TDF will allow disassociation of the preventive method from the type of coitus (wet vs. dry), number of coital acts, the timing of the coitus, the place of coitus, or other sexual activity such as anal or oral sex.

We will study the effectiveness and safety of TDF in a population of young women at high-risk for HIV infection.

1.2 Rational

TDF has been selected for investigation as prophylaxis against HIV in high-risk young women because of its unique pharmacologic profile. In addition to the convenience of being a once daily single tablet, TDF's safety profile is comparable to placebo among HIV infected persons, it has striking anti-HIV potency, and it has low potential for selection of resistant viruses. TDF is cleared from the body by the kidneys and is not metabolized by the liver. Therefore, TDF has limited potential to have pharmacokinetic interactions with other hepatically metabolized drugs. Each of these properties is necessary given the realities of the intended target populations. Moreover, initial prevention studies in simian models have provided encouraging results. Finally, the drug's sponsor, Gilead Sciences, is supportive of investigating the potential use of TDF as a preventive, as well as therapeutic, agent and will provide TDF for the study. Gilead also is willing to make a good faith effort to make TDF available for public health use should it prove to be effective for HIV prevention.

Condoms and potential vaginal microbicides require consistent and correct use during every coital act. This is similar to the situation when barrier methods were the most common forms of nonpermanent contraception. However, when hormonal oral contraceptives were introduced and the preventive method became disassociated with coitus, contraception become easier, popular, and more effective at the individual and population level. If successful, this drug has the potential to do for HIV prevention what oral contraceptives did for pregnancy prevention.

2. STUDY OBJECTIVES

2.1 Primary Objectives

- To assess the effectiveness of TDF 300 mg use by young women at high risk for HIV in countries that will be suitable for the introduction of TDF as a preventive intervention
- To assess the extended safety of TDF 300 mg per day among women who are not HIV infected

Comparing the HIV incidence in the TDF group with that in the control group will assess effectiveness.

Comparing data on changes in kidney and liver function, as well as the frequency of adverse events in the TDF group with that in the control group will assess extended safety.

3. TRIAL DESIGN

This Phase 2 clinical trial will be conducted in compliance with the protocol, FHI SOPs, Good Clinical Practices (ICH/GCP), and the applicable regulatory requirements of the FDA and the site countries.

3.1 Primary Endpoints

The effectiveness endpoint is conversion for antibodies to HIV 1 or 2 as determined by an OMT test and confirmed by an ELISA from a finger prick specimen. Discordant results between the OMT and the ELISA will be tested with Western blot.

Laboratory safety endpoints will include serum creatinine and phosphorus for kidney function, and AST and ALT for hepatic function. Reported adverse events will also be used for clinical evaluation of safety.

3.2 Study Design

This protocol describes a multi-center, randomized, fully-masked, parallel, placebo-controlled study of TDF for pre-exposure prophylaxis of HIV in high-risk women.

One thousand two hundred women from Douala, Cameroon, Ibadan, Nigeria and Tema, Ghana will be randomized 1:1 to once daily use of TDF 300 mg or placebo. Each country will contribute 400 participants to the study. Recruitment of participants will take about 6 months and each participant will be followed for 1 year.

HIV status will be monitored monthly. Participants will be monitored for safety using periodic physical examinations, serial laboratory tests, and adverse event queries.

In order to provide sufficient information for early safety monitoring, we will conduct laboratory tests for kidney and liver function at screening and then at months 1, 3, and then every 3 months thereafter or at their final visit if they withdraw early.

To minimize their risk of contracting HIV infection, women will be counseled monthly to use male condoms every time they have intercourse. Male latex condoms will be provided to participants. Participants converting for antibodies to HIV will be counseled and referred to medical services as appropriate for each country.

3.3 Measures to Minimize Bias

3.3.1 Randomization

The Randomization Manager who is not otherwise involved with the study will develop the allocation sequence using a computer random number generator and randomly varied permuted-blocks. Participants will be assigned their randomization number only after they have qualified for the study and signed the consent form. Randomization will be stratified by study site.

3.3.2 Allocation concealment

Individual assignments will be concealed in sequentially numbered, sealed opaque envelopes. Trained staff will open the envelopes only after participants are properly consented and enrolled. The randomization envelopes will be maintained in a secure office. They will not be available to the study counselors until the immediate moment of randomization. Each randomization envelope will be used only once and maintained in the participant's study files.

3.3.3 Masking

Placebo tablets to match the TDF tablets are available, and contain denatonium benzoate to provide a bitter taste to resemble the active tablets. The participants, field study staff, monitors, statisticians, and FHI staff actively involved in the trial will not know which participants are using TDF and which are using placebo.

3.3.4 Decoding procedure

The FHI Randomization Manager will keep the randomization code. The Manager will provide the code to the FHI Study Clinician if needed to reveal the masking to the Investigator for emergencies. The Manager will provide the code to the DMC during meetings to review the interim data. The Manager will provide the code to the statisticians when they are prepared to unmask the code after completion of the final masked analysis of the data.

3.4 Study Product

Tenofovir (PMPA), 9-[(R)-2-(phosphonomethoxy)propyl] adenine monohydrate) is an acyclic nucleotide analog with activity in vitro against retroviruses, including HIV-1 and HIV-2. Tenofovir is metabolized intracellularly to tenofovir diphosphate (PMPApp), which is a

competitive inhibitor of HIV-1 reverse transcriptase (RT) that terminates the growing DNA chain.

Due to the presence of a phosphonate group, tenofovir has limited oral bioavailability. Tenofovir disoproxil fumarate (tenofovir DF, (9-[(R)-2-[[bis[[(isopropoxycarbonyl)oxy] methoxy]phosphinyl]methoxy] propyl] adenine fumarate)) is a prodrug of tenofovir. Tenofovir DF (TDF) is orally bioavailable in animals and humans and is rapidly converted to tenofovir following absorption.

Treatment-Related Adverse Events (Grades 1-4) Reported in >3% of VIREAD-Treated Patients in the Pooled 902 - 907 Studies (0-24 weeks)[5]

	VIREAD 300 mg	Placebo
Number of patients treated	443	210
Nausea	11%	10%
Diarrhea	9%	8%
Asthenia	8%	8%
Headache	6%	7%
Vomiting	5%	2%
Flatulence	4%	0%
Abdominal Pain	3%	3%
Anorexia	3%	1%

Grade 3/4 Laboratory Abnormalities Reported in ≥ 1% of VIREAD-Treated Patients in the Pooled 902 - 907 Studies (0-24 weeks)[5]

	VIREAD 300 mg	Placebo
Number of patients treated	443	210
Number of patients with Grade 3 or 4 laboratory abnormalities	117 (26%)	78 (37%)
Laboratory abnormalities		
Triglyceride (>750 mg/dl)	37 (8%)	28 (13%)
Creatine Kinase (>782 U/L)	53 (12%)	38 (18%)
Serum amylase (>175 U/L)	21 (5%)	14 (7%)
AST<br>Male >180 U/l<br>Female >170 U/L	16 (4%)	6 (35)
Urine glucose (3+ or 4+)	12 (3%)	6 (3%)
ALT elevation<br>Male >215 U/L<br>Female >170 U/L	10 (2%)	4 (2%)
Serum glucose >250 mg/dL	8 (2%)	8 (4%)
Neutrophil <650/mm^3	6 (1%)	3 (1%)

3.5 Duration of the Study

The study is expected to last a total of 20 months at each site. Enrollment of participants will take 6 months. Each individual participant will be followed for 12 months. Screening will start 1 month before enrollment. The study will remain open for 1 month after the last scheduled follow-up visit to allow follow-up on ongoing AEs and attempts to find participants that have not returned for their scheduled visits.

3.6 Withdrawal Criteria

3.6.1 Individual participants

Participants withdrawn will not be replaced. A participant may be withdrawn early from the study for any of the following reasons:

- She considers it to be in her best interest
- A physician considers it to be in the participant's best interest because of safety reasons and/or for the well-being of the participant

Reasons for withdrawal from the study (and study product) will be recorded on the appropriate DCF. **When early withdrawal from the study occurs, every reasonable effort will be made to assess information relevant to the primary endpoints at the time of discontinuation**. This would include attempts to obtain specimens for HIV testing.

TDF is rated for use in pregnancy as category B: reproduction studies have been performed in rats and rabbits at doses up to 14 and 19 times the human dose based on body surface area comparisons and revealed no evidence of impaired fertility or harm to the fetus due to tenofovir. There are, however, no adequate and well-controlled studies in pregnant women. Because animal reproduction studies are not always predictive of human response, TDF should be used during pregnancy only if clearly needed. Pregnancy is an exclusion criterion for this study. We will use urine pregnancy tests monthly to monitor for pregnancy. Participants who become pregnant will have study drug withdrawn. We will attempt to monitor participants until the conclusion of the pregnancy and the outcome of the pregnancy will be reported to the Gilead Sciences Antiretroviral Pregnancy Registry, as described in the package insert for TDF.

Study product interruption

Treatment with the study product may be interrupted temporarily or permanently for any of the following reasons:

- The participant ran out of supplies for whatever reason (e.g., she missed a visit); in this case the date of interruption is the date of her last product use
- An adverse event
- The Investigator decides to withdraw the study product temporarily in the interests of the safety and well being of the participant

- The participant considers it to be in her best interest not to use the study product
- Pregnancy
- The participant becomes HIV positive

Whenever the study product is interrupted, the information will be noted on the follow-up DCF. For all except the first reason listed above, the date of the interruption is the date on which the Investigator decides to interrupt the treatment and if possible, follow-up should be continued until resolution of the AE. **If possible, participants will be tested for HIV so that the status is determined at the time of product interruption.**

Participants who have had study drug interruption may resume study product use and will be given the study product to which they were originally randomized. **At the time of product resumption the participant will be tested for HIV so that status is determined**.

3.6.2 Study phases

The screening and recruitment phase will be discontinued when the required number of participants has been enrolled. This is anticipated to take 6 months, but may occur earlier or need to be extended.

The scheduled follow-up phase will end 12 months after completion of the recruitment phase. The last woman recruited will finish 12 months of follow-up. Any participant being followed for an AE at the end of the scheduled follow-up period will be followed until her AE resolves or the Investigator determines it is safe to discontinue follow-up.

3.6.3 Study

The study will continue until the planned end of the study, or until such time as:

- FHI decides to discontinue the study. FHI may discontinue a site/investigator due to noncompliance with protocol or regulations
- The IRB decides to discontinue the study

If the study is discontinued before the planned end, each participant remaining in the study at that point will be seen for a final follow-up visit. HIV seroconversions detected through this follow-up visit will be included in the analysis.

3.7 Study Product Accountability

The study sites will receive a supply of study products sufficient for the anticipated number of study participants. All supplies must be stored in a limited access area that is securely locked.

The study site will also procure male latex condoms to dispense for use with the study product.

For purposes of inventory accountability, the study site will not make supplies of the study product available for distribution by any person not part of the study staff or provide these supplies to persons not enrolled in the study.

The study site is required to maintain records of the disposition of each unit of study product received and dispensed, including dates and quantities of distribution to participants, by participant ID number. All unused supplies of the study product must be returned to FHI at the end of the study, or disposed of in a manner specified by FHI.

3.8 Randomization Codes

The FHI Randomization Manager will maintain the trial treatment randomization codes. The code for an individual participant will be made available to the FHI Study Clinician, should the need arise to reveal the code. It is anticipated that the unmasking will occur rarely and then only if knowledge of the treatment group would affect health care for the participant. The Investigator will immediately notify the FHI Project Leader if he suspects that unplanned unmasking has occurred.

3.9 Data Directly Recorded on DCFs

All DCFs used in the field for this study will have data that is directly recorded on them and will be considered source documents. Other source documents include, but are not be limited to, staff journals, medical notes, screening and enrollment logs, laboratory results, pill dispensing records, consent forms, and reimbursement logs.

4.0 SELECTION OF PARTICIPANTS

The target population is women who are sexually active and at high risk of HIV. High risk for the purposes of this study includes having intercourse, on average, three times per week and having had more than three different sexual partners in the last month.

4.1 Eligibility Criteria

To be eligible for inclusion in this study, a woman must be HIV seronegative and:

- Be willing and able to give informed consent
- Be 18 years to 35 years old, inclusive
- Be sexually active (on average, three coitus per week)
- Have had more than three sexual partners in the last month
- Be willing to use study product as directed
- Be willing to adhere to follow-up schedule
- Be willing to participate in the study for up to 12 months
- Not be pregnant, breast feeding, or desire a pregnancy during the 12 months of participation

- Have adequate renal function (serum creatinine <1.5 mg/dL)
- Have adequate liver function (hepatic transaminases (ALT and AST) <3 x ULN
- Have adequate serum phosphorus ($\geq$2.2 mg/dL)
- Be in general good health (no active, serious infections that require parenteral antibiotics, no active clinically significant medical conditions, including heart disease, diabetes, asthma, alcoholism, and cancer)

4.2 Loss to Follow-up Procedures

If a participant fails to appear for a scheduled visit, at least three attempts to contact her should be made over the subsequent 30 days. These attempts should be documented in the participant's study file. After these three attempts, no further efforts need be made to find her, but her file should remain open until study closeout.

If the participant does not return to the study before the study is closed, the Final Disposition Form will be completed at the time of study closeout. The form should indicate that the participant was lost to follow-up. The "lost to follow-up" designation cannot be made for any participant until the closing date of the study.

Participants will be evaluated for efficacy up until the last follow-up visit.

5.0 STUDY PRODUCTS AND TREATMENTS

5.1 Products

Tenofovir DF (GS-4331-05) tablets will be supplied by Gilead Sciences, Inc. Tenofovir DF tablets are almond-shaped film-coated tablets containing 300 mg of TDF. Each tablet contains the following inactive ingredients: microcrystalline cellulose, lactose monohydrate, pregelatinized starch, croscarmellose sodium, and magnesium stearate.

Placebo tablets to match the tenofovir DF tablets contain inactive ingredients listed above and denatonium benzoate to provide a bitter taste to match the active tablets.

TDF or placebo tablets are packaged in white high-density polyethylene (HDPE) bottles with a white child-resistant cap. Each bottle contains 30 tablets. Each bottle also contains a single silica gel canister to protect the product from humidity and fiber packing to protect the product during handling and shipping. TDF or placebo tablets should be stored before dispensing to participants at 25°C; excursions permitted to 15-30°C. Each bottle will be labeled with a randomization number, the protocol number, expiration date, and sponsor address.

To ensure product stability, drugs should not be dispensed in a container other than the container in which they are supplied. Participants will be instructed to return unused study medication in the original container at each study visit.

- The dose is 300mg (one tablet) once daily with a meal or just afterwards.
- The tablets should be swallowed whole.

- TDF works best if there is a constant amount in the bloodstream. It is therefore very important to take the tablets regularly. If a dose is forgotten, take it as soon as remembered.
- The doses should be taken as close as possible to twenty-four hours apart at the same time each day. It is essential that a daily dose is not missed.

5.2 Concomitant Treatments

Nephrotoxic drugs (group A), drugs that slow kidney excretion of drugs (B), drugs directed at immune system modulation (group C), and other retroviral drugs (D) should not be used during the study. The following medications are excluded while patients are participating in the study:

- A. Nephrotoxic agents
    - aminoglycoside antibiotics
    - IV amphotericin B
    - cidofovir
    - cisplatin
    - foscarnet
    - IV pentamidine
    - oral and IV vancomycin
    - oral or IV ganciclovir
    - other agents with significant nephrotoxic potential
- B. Drugs that slow kidney excretion
    - Probenecid
- C. Immune system modulators
    - Systemic chemotherapeutic agents (i.e., cancer treatment medications)
    - Systemic corticosteroids
    - Interleukin-2 (IL-2)
    - Immunomodulators
- D. Other antiretrovirals

Should participants need to start treatment with any excluded concomitant medication, FHI should be informed as soon as possible after initiation of the new medication. In instances where an excluded medication is initiated prior to discussion with FHI, the Investigator must notify the FHI as soon as he/she is aware of the use of the excluded medication.

5.3 Monitoring of Product Compliance

Participants will return the unused tablets and bottle at each month's follow-up visit. Unused tablets will be counted and recorded on the appropriate DCF.

6.0 EFFECTIVENESS AND SAFETY ASSESSMENT

6.1 Effectiveness Parameters

An incident HIV infection is defined as follows:

- The presence of HIV antibodies by OraQuick® rapid test and confirmed by an ELISA from serum collected by a finger prick at a follow-up visit, and
- The absence of HIV antibodies by OraQuick on a sample collected at the previous visit

6.2 Methods and Timing of Effectiveness Parameters

Oral mucosal transudate (OMT) specimens will be tested for HIV-1/2 antibodies at screening, enrollment, and then monthly throughout follow-up using OraQuick. OraQuick HIV-1/2 is a rapid test device that can detect antibodies to the HIV disease within 20 minutes. OraSure Technologies, Inc makes OraQuick.

The OraQuick results will be available 20 minutes after collection. If the OraQuick test is positive, the participant will be told that the test results are preliminary and a finger prick specimen will be collected on filter paper and tested by ELISA. If the OraQuick and ELISA results are discordant, a Western blot from the finger prick specimen will be done. (See section 14.1 for quality control of the OMT HIV sampling.)

6.3 Safety Measurements

We will measure serum ALT, AST, creatinine, and phosphorus as safety measures.

The lab tests called "liver function tests" actually measure the levels of enzymes found in the liver, heart, and muscles. Enzymes are proteins that cause or increase chemical reactions in living organisms. High enzyme levels can indicate liver damage caused by medications, alcohol, hepatitis, or recreational drug use.

- **ALT** (alanine aminotransferase), formerly called serum glutamate pyruvate transaminase or SGPT)
- **AST** (aspartate aminotransferase), formerly called serum glutamic-oxaloacetic transaminase or SGOT)

AST and ALT are sensitive indicators of liver injury. AST is present in the heart, skeletal muscle, brain, and kidney as well as in the liver. AST levels thus rise in myocardial infarct, heart failure, muscle injury, central nervous system disease, and other non-hepatic disorders. AST is relatively nonspecific, but high levels indicate liver cell injury. AST is reliable for routine screening for liver disease. Values > 500 IU/L suggest acute viral or toxic hepatitis and occur with marked heart failure and occasionally with common bile duct stones. The magnitude of the elevation has no prognostic value and does not correlate with the degree of liver damage. Serial

testing provides good monitoring. A fall to normal indicates recovery unless associated with the end stages of massive hepatic necrosis.

ALT is found primarily in liver cells and thus has greater specificity for liver disease but offers little other advantage. In most liver diseases, the AST increase is less than that of ALT (AST/ALT ratio < 1), but in alcohol-related liver injury, the ratio frequently is > 2.

- **Creatinine** is a waste product of protein digestion and a measure of kidney function. High levels are usually due to kidney problems. Clinicians use the creatinine level as most direct sign of how well the kidneys are removing waste products from the body.
- **Phosphorus**, like calcium, is a major component of bones. Low levels of phosphorus for a long period of time can cause damage to bones, nerves and muscles. High phosphorus levels are most often due to kidney failure.

6.4 Methods and Timing of Safety Measurements

A blood sample will be drawn from participants for AST, ALT, creatinine, and phosphorus testing at screening (-1), and months 1, 3, 6, 9, and 12 during follow-up.

7.0 ADVERSE EVENTS AND REPORTING REQUIREMENTS

7.1 Adverse Events

Study participants will be provided with contact information and instructions to contact the Investigator or designee to report any adverse events (AEs) they may experience, except for life-threatening events, for which they will be instructed to seek immediate emergency care. Based on the results of the Investigators' assessment, s/he will recommend either continuation or discontinuation of product use. The Investigator also may prescribe or recommend the use of medications or other preparations to treat the AE.

Where feasible and medically appropriate, participants will be encouraged to seek medical care where the site study clinician is based, and to request that the clinician be contacted upon their arrival. With appropriate permission of the participant, records from all non-study medical providers related to AEs will be obtained and required data elements will be recorded on study case report forms. All participants reporting an AE will be followed clinically, until the AE resolves (returns to baseline) or stabilizes.

Study site staff will document on study DCFs all AEs reported by or observed in enrolled study participants regardless of severity and presumed relationship to study product. Site staff also will report information on all AEs to their IRB in accordance with US Federal regulations and local IRB requirements.

An AE is any unfavorable and unintended sign, symptom, or disease temporally associated with the use of a medicinal product, whether or not considered related to the medicinal product. Pre-existing events, which increase in frequency or severity or change in nature during or because of use of a drug in human clinical trials, will also be considered as adverse events. AEs may also

include pre- or post-treatment complications that occur as a result of protocol-mandated procedures (e.g., invasive procedures such as biopsies).

Any medical condition or clinically significant laboratory abnormality with an onset date before the first date of study drug administration is considered to be pre-existing, and should be documented on the DCF noting that it is pre-existing.

Any AE (i.e., a new event or an exacerbation of a pre-existing condition) with an onset date after study product administration up to the last day on study (including the follow-up) should be recorded as an AE on the appropriate DCF.

The Investigator must determine the severity of the AE and document it on the appropriate DCF (AE Form). Each adverse event that the participant is aware of should be graded for severity using the following scale:

- **Very slight:** participant was aware of the adverse event and it disappeared soon after or it was on and off.
- **Mild:** participant was aware of the adverse event all of the time, but was still able to do all activities.
- **Moderate:** the participant had to discontinue some activities due to the adverse event.
- **Severe:** the participant was incapacitated by the adverse event and unable to perform normal activities.

An AE **does not** include:

- Medical or surgical procedures (e.g. surgery, endoscopy, tooth extraction, transfusion); the condition that leads to the procedure is an adverse event.

- Pre-existing diseases or conditions present or detected prior to start of study drug administration that do not worsen.

- Situations where an untoward medical occurrence has not occurred (e.g. hospitalization for elective surgery, social and/or convenience admissions).

- Overdose of either study drug or concomitant medication without any signs or symptoms unless the participant is hospitalized for observation.

7.2 Relationship of adverse event to study product

The Investigator must determine the relationship of the AE to the product under investigation and document on the appropriate DCF (AE Form). For each AE, an assessment of the relatedness to the test agent should be made using the following scale:

- **Unrelated**: Onset of the AE had no reasonable temporal relationship to administration of the study product or a causal relationship to administration of the study product is biologically implausible or the event is attributed to an alternative etiology.

- **Possibly Related**: Onset of the AE has a reasonable temporal relationship to study product administration and a causal relationship is not biologically implausible.
- **Probably Related**: Onset of the AE has a strong temporal relationship to administration of the study product that cannot be explained by the participant's clinical state or other factors and a causal relationship is not biologically implausible.
- **Definitely Related**: Onset of the AE shows a distinct temporal relationship to administration of the study product that cannot be explained by the participant's clinical state or other factors or the AE occurs on re-challenge or the AE is a known reaction to the product or chemical group or can be predicted by the product's pharmacology.

These criteria in addition to good clinical judgment should be used as a guide for determining the causal assessment. If it is felt that the event is not related to study drug therapy, then an alternative explanation should be provided.

7.3 Reporting adverse events

All AEs occurring during the study will be reported on the appropriate DCF (AE Form). The Investigator must provide on this form information on symptoms, time of onset, severity, frequency, product-relatedness, action(s) taken, and participant outcome. FHI's Study Clinician may request additional information from the site if additional information is needed to evaluate the AE.

7.4 Serious Adverse Events

A **serious adverse event** (SAE) for a drug or biologic product or medical procedure is defined as any experience that suggests a significant hazard, contraindication, side effect, or precaution. A serious adverse event includes any adverse experience that results in any of the following outcomes:

- Death
- A life-threatening adverse drug experience
- Inpatient hospitalization or prolongation of existing hospitalization
- A persistent or significant disability/incapacity
- A congenital anomaly/birth, or
- An important medical event that, based on medical judgment, may jeopardize the patient or subject and may require intervention to prevent one of the outcomes listed above.

Examples of such events are:

- Intensive treatment in an emergency room or at home for allergic bronchospasm
- Blood dyscrasias or convulsions that do not result in hospitalization
- Development of drug dependency or drug abuse.

Clarification of SAEs:

- Death is an outcome of an adverse event, and not an adverse event in itself.
- All deaths, regardless of cause, must be reported for participants on study and for deaths occurring within 30 days of last study product use or within 30 days of last study evaluation, whichever is longer.
- "Occurring at any dose" does not imply that the participant is using study product at the time of the event. Product use may have been interrupted temporarily prior to the onset of the SAE, but may have contributed to the event.
- "Life-threatening" means that the participant was at immediate risk of death from the event as it occurred. This does not include an event that might have led to death, if it had occurred with greater severity.
- Complications that occur during hospitalizations are AEs. If a complication prolongs hospitalization, it is a SAE.
- "Inpatient hospitalization" means the participant has been formally admitted to a hospital for medical reasons, for any length of time. This may or may not be overnight. It does not include presentation and care within an emergency department unless the participant is "admitted".
- The Investigator should attempt to establish a diagnosis of the event based on signs, symptoms and/or other clinical information. In such cases, the diagnosis should be documented as the AE and/or SAE and not the individual signs/symptoms.

7.5 Reporting Serious Adverse Events

All serious adverse events must be reported to FHI ***within 24 hours*** of the study site becoming aware of the problem. The Investigator should complete a FHI SAE Report Form and fax it simultaneously to:

- ***Dr. Kavita Nanda (the FHI Study Clinician)***
- ***Mr. David Borasky (The FHI protection of Human Subjects Committee Representative)***

Family Health International
Telephone: 919-544-7040
Fax: 919-544-7261
Email: dborasky@fhi.org and knanda@fhi.org

In cases in which a SAE Report Form cannot be faxed within 24 hours, the Investigator may report the SAE via telephone or e-mail; however, a SAE Report Form must be completed as soon as possible after the informal report. Based on the preliminary information, the FHI Study Clinician will complete an interim SAE Report Form and forward it to the PHSC Representative for processing. The Study Clinician will make sure to update this information as soon as he/she receives a complete SAE Report Form from the Investigator.

The FHI Study Clinician and FHI Chief Medical Officer will complete the FHI SAE Review Form within 5 days of receipt of the SAE Report Form (including interim Report Form) from the site.

If the SAE is unexpected, and related to the use of an investigational product, the FHI Study Clinician will alert the FHI Regulatory Associate immediately of receipt of the SAE Report Form that an SAE has occurred that may be reportable to the regulatory agency. The FHI Study Clinician will process and report all SAEs to the Regulatory Associate and PHSC.

The Investigator will be responsible for proper reporting of all SAEs to the local IRB, to the FHI Study Clinician and PHSC Representative as specified in the protocol.

7.6 Adverse Event Management

See Appendices 1, 2, and 3 for more details.

7.6.1 Grades 1 and 2 laboratory abnormality or clinical event (except serum creatinine elevation)

- Continue study drug at the discretion of the Investigator.

(See Appendix 2 for details)

7.6.2 Grade 3 laboratory abnormality or clinical event (except serum creatinine elevation)

- For a Grade 3 laboratory abnormality or clinical event, study drug may be continued if the event is considered to be unrelated to study drugs.
- For a Grade 3 clinical event, or laboratory abnormality confirmed by repeat testing, that is considered to be related (any degree of relatedness) to study drugs, study drugs should be withheld until the toxicity returns to at least Grade 2, at which point the patient can be re-challenged with study drug.
- If toxicity recurs to Grade 3 following re-challenge with study drugs and is considered related to the study drug, then the study drug should be permanently discontinued and patients managed according to local practice.
- Recurrence of events considered unrelated to study drug do not require permanent discontinuation of the study drug.

(See Appendix 2 for details)

7.6.3 Grade 4 laboratory abnormality or clinical event (except serum creatinine elevation)

- For a Grade 4 clinical event, or laboratory abnormality confirmed by repeat testing, that is considered related to any of the study drugs, all study drugs should be permanently discontinued and patients managed according to local practice. The patient should be followed as clinically indicated until the event resolves to baseline, or is otherwise explained, whichever occurs first.

- For a Grade 4 laboratory toxicity that is not confirmed by repeat testing should be managed per algorithm for the new toxicity grade.

- Study drug may be continued without dose interruption for a Grade 4 laboratory abnormality or clinical event considered unrelated to the study drug.

(See Appendix 2 for details)

7.6.4 Grade 1 serum creatinine elevation

For the purposes of serum creatinine, the baseline value is defined as the last value prior to the administration of the first dose of study drugs. This value must be obtained from the laboratory. Serum creatinine values = 0.5 mg/dL above baseline should be confirmed by repeat testing within 3 calendar days of receipt of results and before study drugs discontinuation, unless such a delay is not consistent with good medical practice.

- For Grade 1 serum creatinine elevations, patients may continue study drug, but it is recommended that participants be monitored weekly until the serum creatinine returns to the original baseline value or $\leq$0.3 mg/dL from baseline.

(See Appendix 3 for details)

7.6.5 Grades 2-4 serum creatinine elevations

- Study drug should be permanently discontinued in the event that repeat testing of serum creatinine confirms a value equal to or higher than 2.1 mg/dL. The patient should be followed at periodic intervals not less than weekly, until the serum creatinine falls to within 0.3 mg/dL of the baseline value.

(See Appendix 3 for details)

7.7 Social Risk Events

No social risk events are expected from participation in this study. However, if the Investigator learns of a social risk event, he will report the event on the Social Risk Event Form.

8.0 STUDY VISIT SUMMARY

The participants will make visits to study sites for one screening, one enrollment, and 12 monthly follow-up visits. A table summarizing study procedures is available in Appendix 4.

8.1 Screening Visit

The study, including potential risks and benefits of participation, will be explained to prospective participants during the screening visit and before any procedures are begun. This will include a review of the study purpose and procedures. Each woman will be asked to sign (or provide other mark) a consent form for the screening procedures, and will be given a signed copy of the consent form to take home.

All volunteers will be assigned an ID number (for the purpose of tracking their lab tests), and evaluated to determine their eligibility according to the participation eligibility criteria.

The following will be done:

- An interview with the woman to make sure she understands the study schedule and is willing to comply with study requirements
- Screening consent obtained
- Screening questionnaire
- A urine pregnancy test
- Pre-test and post-test HIV counseling
- OMT sample for HIV testing and a finger prick specimen if needed for confirmation
- Blood drawn for kidney and liver functions
- The women will be asked to return in 4 weeks for the enrollment visit, when they will receive the results of the blood tests.
- All women will be instructed in proper condom use.

8.2 Enrollment Visit

If the woman meets all eligibility criteria, she will receive a detailed explanation of study procedures, and counseled in the importance of returning for follow-up. If the screening test for HIV was negative, the potential participant will be re-screened with OraQuick® on the day of enrollment. She will be asked to sign an enrollment consent form, and will be given a signed copy to take home. She will then be randomized.

The following will be done at the enrollment visit:

- An interview with the woman to make sure she understands the study schedule and is willing to comply with study requirements
- Enrollment consent obtained
- Enrollment questionnaire
- A urine pregnancy test
- Pre-test and post-test HIV counseling
- OMT sample for HIV testing and finger prick specimen if needed for confirmation
- Randomization
- Drug and condom distribution

Counseling
The counselor will review study product use, and methods of HIV/STI transmission and prevention. The use of condoms with all partners, including primary sexual contacts, will be emphasized. Staff will also stress that this is a controlled trial of a product of unknown effectiveness in the prevention of HIV transmission and that condoms should be used for all sexual contacts with all partners. This information will be repeated at each follow-up counseling session.

Study Product
Each participant will receive a supply of TDF or placebo and offered condoms. Participants will receive supplies of condoms in quantities anticipated to last until the next visit. A participant can return between scheduled visits to obtain more condoms if needed. A product-dispensing log will be maintained by the clinic and field staff to track all product distribution.

Pelvic examination
The pelvic examination will only be done if the participant has symptoms or a problem that needs to be investigated. If a wet mount is positive for *T. vaginalis,* yeast or bacterial vaginosis, the woman can be enrolled while starting treatment. Any other diagnosed curable STD can be treated and the woman enrolled. STD treatment will be recorded on the appropriate DCF.

8.3 Follow-up Visits

Each participant will have a follow-up visit at a study site convenient for her and at the study clinic (months 1, 3, 6, 9, and 12). Study sites distributed throughout the study cities will be chosen as follow-up sites. Each outreach worker will be assigned to a study site and will use the site as a base of operation. The outreach worker is responsible for monthly counseling, interviewing, re-supplying the participants with products, and, if necessary, home visits to ensure that each participant completes the study. Activities at the monthly visit include:

- A questionnaire will be administered to collect information on use of product and condoms
- Participants will be counseled about product and condom use
- Participants will be re-supplied with study product and offered condoms in an amount sufficient to last until the next follow-up visit
- A pregnancy test
- Participants will provide an OMT specimen for HIV testing and a finger prick specimen if needed for confirmation

Study participants will be met at the clinic by the outreach worker for visits at months 1, 3, 6, 9, and 12 so that venous blood can be taken for the safety parameters.

If at any visit, the staff person or the study participant thinks that an examination is necessary, the participant will be referred to the study clinic for an examination.

8.4 Interim Contacts and Unscheduled Visits

Participants may make interim contacts and unscheduled visits at the participant's request or as deemed necessary by the Investigator at any time during the study. All interim contacts and unscheduled visits will be documented in the participants' study records and on applicable DCF.

When an interim contact or unscheduled visit occurs in response to AEs experienced by study participants, study staff will assess the reported event clinically and provide or refer the participant to appropriate medical care.

Study participants will be instructed to contact their study clinic in the event of any adverse events (AE) except for life-threatening AE, for which participants will be instructed to seek immediate emergency care. The AE will be reported on the appropriate DCF.

Interim visits are distinguished from adverse event visits in that no health related reaction, effect, or abnormality is reported. Some examples of reasons for an interim visit are:

- To get more product or condoms
- To ask questions of study staff
- To discuss problems with study compliance
- Interim examination - examination requested but no complaints or symptom

9.0 STATISTICAL SUMMARY

9.1 Study Size Justification

9.1.1 Effectiveness outcome

This study is designed to have 90% power to conclude with 95% confidence that TDF reduces the rate of HIV infection by 50% (i.e., power to obtain a one-sided 95% upper confidence limit for the rate ratio that is less than 0.5) if the true rate of reduction due to TDF is at least 83%. Recruiting 1,200 participants, and following each for 12 months, should provide the required number of incident HIV infections (30), assuming that HIV incidence in the placebo group is no less than 5/100 person-years, and follow-up is at least 80% at 12 months.

9.1.2 Safety outcomes

We will assess safety outcomes by treatment group (pooled across sites), as well as in each site-by-treatment group cohort. Site-level information will be assessed to provide each country's public health sector with data to make informed safety decisions in their own populations. As demonstrated in Table 1, precise estimates of 12-month event probabilities should be obtained at the site-level using the chosen study size. Furthermore, the study size is adequate to observe toxicity outcomes that occur at a modest rate of 5%; the probability that five or more such events are observed among a cohort of 200 participants is greater than 95%.

Table 1: 95% Confidence intervals corresponding to 12-month safety event probability estimates, assuming 20% loss to follow-up.

If estimated 12-month cumulative probability is equal to…	Then the 95% confidence interval is…	
	Site-level (N=200)	Pooled (N=600)
1%	(>.000, .024)	(.002, .018)
2%	(>.000, .040)	(.008, .032)
3%	(.005, .055)	(.016, .044)
5%	(.018, .082)	(.032, .068)
10%	(.056, .144)	(.075, .125)

9.2 Analysis Plan Summary

A detailed analysis plan that covers both the final analysis and the planned interim analyses will be developed and approved prior to initiation of the study. The following is a summary of the planned analyses. Any deviations to be made from this summary plan will be documented in the detailed analysis plan.

All primary and most secondary analyses will be conducted on an intent-to-treat basis. However, in order to assess the effectiveness and safety of TDF among those participants who complied with key aspects of the study protocol and to evaluate the potential impact of treatment on loss to follow-up, various additional analysis populations may be defined in the detailed analysis plan. Any key decisions regarding the timing of outcomes, the appropriateness of test statistics or model assumptions, or any other statistical issues will be made in a masked review of the data (i.e., masked to the true treatment assignments). Unmasking with respect treatment assignment will only be done for the final interpretation of the results, unless otherwise required by the data monitoring committee (DMC) (see section 10.3).

9.2.1 Analysis of effectiveness outcomes

All primary analyses will be conducted on an intent-to-treat basis. Incidence rates of HIV infection in the two treatment groups will be calculated, and the effect of TDF on the incidence of infection will be determined by calculating an upper 95% confidence bound for the rate ratio (TDF *versus* placebo). This upper confidence bound will be compared to 0.5 to test the hypothesis that the reduction in the HIV infection rate due to TDF is at least 50% (i.e., a one-sided test of superiority with $\alpha = 0.05$). The estimated rate ratio and 95% confidence bound will be obtained using Cox proportional hazards regression, controlling for site, if the model assumptions are found to be appropriate. Otherwise, crude incidence ratio estimates and confidence bounds will be obtained based on the ratio of the numbers of new cases to the total lengths of treatment exposure.[6] Time to HIV infection, in days, will be computed as the difference between the date of HIV infection, estimated using the midpoint of the study interval in which the first positive HIV test was obtained (see Section 6.1) and the enrollment date, plus one. Potential prognostic factors identified prior to study initiation will be adjusted for in supporting analyses of incident infections. Likewise, supporting analyses will evaluate the treatment effect across sites by introducing treatment-by-site interaction terms to the proportional hazards regression model.

9.2.2 Safety outcomes

Primary laboratory safety endpoints include serum creatinine and phosphorus for kidney function, and AST and ALT for hepatic function. Here, laboratory safety events for creatinine are defined as the occurrence of grade 2-4 serum elevations at any time during follow-up. Laboratory safety events for phosphorus, AST, and ALT are defined as the occurrence of grade 3-4 laboratory abnormalities during follow-up. Twelve-month cumulative event probabilities for each of these endpoints will be estimated based on the time to the first event using the Kaplan-Meier method and Greenwood's formula for standard errors.[7] Differences in the estimated 12-month event probabilities between treatment groups will be presented along with 95%

confidence intervals. In addition, changes in lab results between baseline and follow-up visits will be summarized descriptively (e.g., tabulating means, medians, standard deviations, ranges, and number of participants with abnormal values), by treatment group and site.

The frequency and percentage of adverse events will by tabulated by body system. Listings of all AE's will include information on duration, outcome, severity, and relation to product use. Differences between treatment groups in the frequency of body-system level AE's will be evaluated using exact, two-sided 0.05 level tests, and approximate 95% CIs stratified by site.

9.3 Interim Analysis Plan Summary

FHI will conduct interim analyses of safety data at 6 and 12 months after enrollment begins (see section 10.3). Each interim analysis will include comparability of masked treatment groups at enrollment, compliance with intervention, discontinuation rates, and intervention-associated morbidity.

In addition to providing safety data summaries, a biostatistician not otherwise associated with the study will perform an interim study size re-assessment calculation prior to the first interim safety analysis. S/he will make recommendations to the DMC regarding the potential need to increase study size if HIV infection or follow-up rates are lower than anticipated (a reduction in the total study size on grounds of excess power will not be permitted). Any such recommendations will be based on the infection rate pooled across both treatment groups, and not on observed differences between treatment groups; hence, no type I error rate adjustment will be required when evaluating the primary hypothesis test specified in section 9.2.1.

10.0 MONITORING PLAN SUMMARIES

10.1 Clinical Monitoring Plan

Site visits by the FHI clinical monitors will be made in accordance with FHI policy and SOPs. The purpose of the monitoring is to assure the quality and accuracy of data collected on the DCF and entered in the database, and to determine that all regulatory requirements surrounding clinical trials are met.

The Investigator will allow the clinical monitors, the regulatory agencies, and FHI designated persons to inspect study documents (e.g., consent forms, drug distribution forms, DCF) and pertinent clinic records for confirmation of the study data.

Various authorized individuals may visit the study site to audit the progress of this study (e.g., FHI personnel, product manufacturer personnel, regulatory personnel). A site visit log will be maintained at the study site in which all site visits made by authorized individuals are recorded. All clinical records and the DCF for the participants enrolled in this study will be made available for review by these authorized individuals.

Before the study begins, the clinical monitors will conduct an evaluation visit at each site to determine the suitability of the site for the research study. In addition, there will be a study

initiation visit (study start-up), quarterly monitoring visits (to track study progress) and a close-out visit. The overall responsibility of the monitors is to ensure that the study is being conducted according to the protocol, FHI SOPs, ICH/GCP and applicable regulatory requirements.

A detailed monitoring plan will be developed for this study and will be used by all clinical monitors. This plan will specify the responsibilities and qualifications of the identified clinical monitors, back-up provisions, in-house monitoring procedures, and site monitoring visit procedures. All monitoring visits will be documented.

10.2 Safety Monitoring Plan

The Study Clinician will review all abnormal safety parameter AEs (creatinine, phosphorus, ALT, or AST). The Study Clinician will review quarterly a cumulative summary of the AEs reported. The Study Clinician will review (in compliance with FHI SOP) all SAEs.

10.3 Data Monitoring Committee

A Data Monitoring Committee (DMC) will review the safety profile of this study while the study is ongoing. The committee will convene 6 and 12 months after start of enrollment. The DMC will examine the safety results of the trial. The results broken out by the treatment groups will remain masked for the FHI personnel.

No formal stopping rules will be used by the DMC for safety outcomes. Rather, a clinical assessment will be made to determine if the nature, frequency, and severity of adverse effects associated with TDF warrant the early termination of the study in the best interests of the participants.

FHI has no a priori intent to consider early termination of the study based on effectiveness so that the extended safety data can be collected. Data on safe use in non-HIV infected people for at least one year is as important as the HIV outcome.

11.0 DATA MANAGEMENT PLAN SUMMARY

A detailed data management plan will be written prior to study initiation. It will be modified if significant changes are required, in order to document how data were handled in the study. The following is a brief summary of the plan.

Study sites will capture clinical data on paper DCFs. After each patient visit, sites will enter data directly from the source documents into the eDCF using InForm software. Once the data have been entered and submitted, the information is encrypted and transmitted over the Internet to a PharmaLinkFHI database server. The server runs the pre-programmed validation checks against the new data and immediately returns any automated queries to the site. An audit trail of each change to the data is captured within the system.

FHI clinical monitors will perform the in-house clinical review of the participant data remotely for clinical consistency, outliers, AEs, and other abnormalities. Based on this review, manual queries are entered into InForm and are immediately available to sites for reply. All queries are automatically tracked in the database for auditing purposes using a full audit trail.

Throughout the study, the PharmaLinkFHI data manager will review the data on an aggregate level to identify potential areas requiring further clarification and look for data quality trends. This review supplements the remote page-by-page eDCF review carried out by the FHI monitor, and allow PharmaLinkFHI to identify potential data inconsistencies that only appear when one value is compared to the other values that have been collected during the study. Regular aggregate data review will allows PharmaLinkFHI to substantially decrease the time to providing the data set for analysis by proactively addressing many data issues that usually only appear at the end of a study when the summary data displays are reviewed.

PharmaLinkFHI will perform adverse event coding using MedDRA, which is a standard dictionary and concomitant medication using WHO. FHI will review and approve all coding prior to final data base closure.

12.0 PROTOCOL VIOLATIONS

Emergency departures from protocol that eliminate an apparent immediate hazard to participants and are deemed crucial for the safety and well being of that participant may be instituted for that participant only – the Investigator will contact FHI as soon as possible. The Investigator will notify the IRB and FHI Project Leader in writing as soon as possible and document on the DCF reasons for deviation and ensuing events.

12.1 Protocol Violations

- Omission or inadequate administration of informed consent
- Inclusion/exclusion errors, including legal age limit
- Treatment errors: no treatment or incorrect treatment (including dose or regimen, expired product), participant compliance problems
- Missing or incorrectly timed study procedures and assessments
- Forcing a participant to enter or remain in the study
- Participants who should have been discontinued from the study due to protocol criteria, but were not

13.0 STUDY DOCUMENTS

13.1 Study Initiation

The following documents will be in place and monitored by a Clinical Monitor at the study site before any potential participants are contacted.

- Investigator’s Brochure

- FDA Form 1572
- Signed protocol and amendments
- Sample DCFs
- Financial disclosure forms for Investigator and Sub-Investigator
- Information given to participants
    - Consent forms
    - Other written information
    - Advertisements
- Financial records
- Signed agreements between involved parties
- IRB approval
- IRB composition
- Regulatory authority approval
- CV for Investigator, Sub-Investigator, and Site Coordinator
- Laboratory test normal values
- Lab accreditation and lab supervisor's CV
- Instructions for handling of investigational product and trial related materials
- Shipping records
- Decoding procedures for masked trials
- Site Visit Log

13.2 Study Conduct

During the study the following documents will be in place and periodically monitored by a Clinical Monitor at the study site. Revision of documents will be made if relevant.

- Revisions to the Investigator's Brochure
- Revisions to the FDA form 1572
- Revision to the Financial Disclosure forms
- Revision to the protocol and amendments
- Revisions to the DCFs
- Revisions of information given to participants
    - Consent forms
    - Other written information
    - Advertisements
- IRB approvals
- IRB composition changes
- Regulatory authority approvals
- CV for Investigator, Sub-Investigator, and Site Coordinator
- Updates of laboratory test normal values
- Documentation of investigational product and trial related materials shipment
- Relevant communications other than site visits
- Signed consent forms
- Source documents

- Copies of completed DCFs
- Copies of documentation of DCF corrections
- Notification of Investigator to FHI of SAEs
- Notification of Investigator to local IRB and Regulatory Authorities
- Notification by FHI or Sponsor to Investigator of safety information
- Interim or annual reports to IRB, Regulatory Authorities, and FHI
- Participant screening log
- Participant ID code list
- Participant enrollment log
- Investigational products accountability documents
- Staff signature list
- Site Visit Log
- Progress Reports to FHI

13.3 Study Completion

After completion of the study, all of the documents in 13.1 and 13.2 should be in the file together with the following.

- Investigational products accountability documents
- Documentation of products destruction (if destroyed at site)
- Complete participant ID code list
- Final report by Investigator to IRB, Regulatory Authorities, and FHI

13.4 Site Record Retention and Access to Documents at the Site

The signed original informed consent documents for each participant, pink copies of DCFs, and originals of all study documentation (e.g. drug inventory forms, participant clinic records, original laboratory reports, staff journals, etc.) will be retained by the Investigator. **No records may be destroyed without written permission from FHI.**

The investigator may be subject to a field audit by FHI to validate the participation of study participants and to verify the data reported on the DCFs. This audit could occur while the study is in progress or several years after the study is completed. All of the participants' records and other study documentation must be filed and accessible on short notice (3-5 days) during the study and subsequent retention period.

14.0 QUALITY CONTROL AND QUALITY ASSURANCE

14.1 Quality Control of the OMT Sampling

Each time that a scheduled blood specimen is drawn from the participants for the safety parameters, an OraQuick HIV test will be performed on the serum. OraQuick can be conducted using whole blood or serum as well as OMT. We will compare the results of the blood specimen

with the OMT specimen at screening, 1, 3, 6, 9, and 12 months during follow-up. Discrepancies will be addressed by testing with ELISA and if discordant, Western blot.

14.2 Quality Assurance

A Quality Assurance Associate from FHI will conduct a site visit and evaluation after at least 5 months of study conduct and then again about 6 months before the projected end of the study. The QA Associate will evaluate study systems for quality control, monitoring of the study, documentation of the study, and overall control of the study.

15.0 ETHICS AND RESEARCH INTEGRITY

15.1 Institutional Review Board Review and Approval

Before contact with potential participants, the study must be approved in writing by the Institutional Review Board at the site and FHI's Protection of Human Subjects Committee in accordance with FDA regulations (21 CFR Part 56). The study will be conducted in accordance with all conditions of approval by the IRBs. The sites will obtain written reapproval of the research by the IRBs at least annually and forward a copy of the review to FHI.

15.2 Informed Consent

No participant may be admitted into this study until the Investigator has obtained her legally effective informed consent. An Investigator shall seek such consent only under circumstances that provide the prospective participant with sufficient opportunity to consider whether or not to participate in the study. Informed consent must be obtained without coercion, undue influence, or misrepresentation of the potential benefits or risks that might be associated with participation in the study.

Informed consent encompasses all oral or written information given to the participant about the study and the study materials. This includes the consent form signed by the participant, recruitment advertising, and any other information provided to the participant. All such information that is given to the participant will be in a language that is understandable to her. The information will not include any language in which the participant is made to waive any of her rights or which releases or appears to release the Investigator, the Investigator's institution, or FHI from liability for negligence.

Informed consent will be documented by the use of a written consent form that is signed by the participant (or the participant's mark if she cannot sign). A copy of the signed consent form will be given to each participant. The original signed consent form for each participant will be kept in the participant's study files separate from the DCFs. The consent form must include each of the basic and additional elements of informed consent described in 21 CFR Part 50.25 and must describe each of the risks or discomforts to the participant that have been identified by FHI as reasonably foreseeable.

15.3 Participant Confidentiality

The confidentiality of all participants enrolled into this study will be protected to the fullest extent possible. FHI staff, FDA personnel, or other individuals authorized in writing by FHI may audit participant's clinic records. However, study participants should not be identified by name on any DCF or on any other documentation sent to FHI and will not be reported by name in any report or publication resulting from data collected in this study.

15.4 Research Integrity

Concern in the United States about the quality of biomedical and behavioral research has led to the establishment of regulations and guidelines for handling the allegations of scientific misconduct. As a recipient of U.S. government funding, FHI is required to develop policies and procedures related to scientific misconduct that conform to these regulations. The regulations define "misconduct" in science as a "fabrication, falsification, plagiarism, or other practices that seriously deviate from those that are commonly accepted within the scientific community for proposing, conducting, or reporting research. It does not include honest errors or honest differences in interpretations or judgments of data" (42 CFR 50.102.)

FHI has specific obligations under these regulations with respect to the handling of information related to scientific misconduct that may come to its attention. In addition, the regulations require that FHI establish procedures related to initiating an inquiry, pursuing an investigation, and informing a specified circle of authorities should the situation warrant it. FHI will pursue all allegations of misconduct in research or questionable academic conduct that may raise legitimate suspicions of misconduct, and will conduct inquiries and investigations to resolve questions regarding the integrity of research. In conducting inquiries during an investigation, FHI will focus on the substance of the issue and will be vigilant not to permit personal conflicts between colleagues to obscure the facts.

16.0 PUBLICATION POLICY

All information concerning the drug supplied by FHI to the Investigator and not previously published is considered confidential.

The initial publication from this study will be the aggregate multicenter results focusing on the primary endpoints of TDF safety and HIV prevention effectiveness. Subsequent secondary analyses and manuscripts, including any single center data, will be agreed upon by members of the protocol team and reviewed by FHI. All suggestions for the manuscript will be returned to the first author by FHI within 15 working days of receiving it.

For publication of the main findings of this study, one person from each site, the Project Leader and Lead Statistician from FHI will be considered as an author (a total of 5 persons). Other key contributors to the research will be acknowledged as part of the TDF Phase 2 Research Team.

17.0 ADDITIONAL INVESTIGATOR RESPONSIBILITIES

In addition to the previously described responsibilities, the Investigator is responsible for signing and dating the cover page of this study protocol. The signed and dated original must be submitted to FHI before protocol implementation, and the Investigator at the site must maintain a copy with the study files.

All protocol amendments must be signed and dated by the Investigator. The signed and dated original must be submitted to FHI before implementation of the amendment, and the Investigator at the site must maintain a copy. Amendments must be approved by FHI and the IRB before implementation.

The Investigator will provide FHI with a curriculum vitae (CV) for himself showing the education, training, and experience that qualifies him as an expert in the area of clinical investigation specific to the product under investigation and his affiliation with the site at which the study is being conducted. CVs also must be provided for all individuals listed as Sub-Investigators on the Form FDA-1572 and the Site Coordinator showing the education, training, and experience that qualifies them for their role in the study, and their affiliation with the study site. If and when any personnel listed on the 1572 changes, the Investigator will notify FHI and provide a CV for any new staff on the form.

The Investigator will supply FHI with copies of the current license and/or laboratory certification (such as the Clinical Laboratory Improvement Act [CLIA] certification) of any laboratory used for the study. The Investigator is responsible for obtaining any updates to these documents and sending them to FHI in a timely fashion. This includes documentation of the normal ranges of the laboratory tests used by the laboratory. The Investigator will ensure that appropriate health care or referral is provided for the study participants throughout the study. Copies of all these documents must be maintained at the site.

Throughout the course of the study, the Investigator will prepare and submit to FHI whatever reports are required and detailed in the Subcontract Agreement to be signed prior to initiating the study. These reports summarize the accomplishments of the assignment and usually include the following:

- all changes in the research activity; and
- all unanticipated problems involving risks to participants or others.

18.0 REFERENCES

1. De Clercq E, Sakuma T, Baba M, et al. Antiviral activity of phosponylmethoxyalkyl derivatives of purine and pyrimidines. Antiviral Res 1987;8:261-272.

2. Gilead Sciences Inc. Investigator's Brochure: Viread. Eighth Edition. October 31, 2002.

3. Tsai C-C, Follis Ke, Sabo A, et al. Prevention of SIV infection in macaques b ®-9-(2-phosphonylmenthoxypropyl) adenine. Science 1995;270:1197-1199.

4. Miller C, Rosenberg Z, Bishofberger N. Use of topical PMPA to prevent vaginal transmission of SIV [oral presentation]. 9th International Conference on Antiviral Research. 1996 May 19-24; Urabandai, Fukushima, Japan.

5. Gilead Sciences Inc. VIREAD Full Prescribing Information. October 1, 2001.

6. Rothman, K.J. and Greenland, S.. *Modern Epidemiology*, 2nd edition. Philadelphia: Lippincott-Raven. 1998.

7. Collett, D. *Modeling Survival Data in Medical Research*. London: Chapman and Hall. 1994.

APPENDIX 1: COMMON TOXICITY GRADING SCALE

GILEAD SCIENCES modified from NIAID

Hematology	Grade 1	Grade 2	Grade 3	Grade 4
Hemoglobin	8.0-9.4 g/dL	7.0-7.9 g/dL	6.0-6.9 g/dL	< 6.0 g/dL
Absolute Neutrophil Count	1000-1500/mm^3	750-999/mm ^{3}	500-749/mm ^{3}	< 500/mm3
Platelets	75,000-100,000/mm^3	50,000-74,999/mm ^{3}	25,000-49,999/mm ^{3}	< 25,000/mm^3 or diffuse petechiae
Prothrombin time	15 - 19 sec.	20 - 22 sec.	23 - 45 sec.	> 45 sec.
Activated partial thromboblastin time	37 - 61 sec.	62 - 86 sec.	87 - 111 sec.	> 111 sec.
Serum Chemistries				
Sodium	130-< 131 mEq/L or 148-150 mEq/L	123-129 mEq/L or 151-157 mEq/L	116-122 mEq/L or 158-165 mEq/L	< 116 mEq/L or > 165 mEq/L
Potassium	3.0-3.3 mEq/L or 5.6-6.0 mEq/L	2.5-2.9 mEq/L or 6.1-6.5 mEq/L	2.0-2.4 mEq/L or 6.6-7.0 mEq/L	< 2.0 mEq/L or > 7.0 mEq/L

Appendix 1: Gilead Sciences Modified NIAID Common Toxicity Grading Scale (Continued)

Serum Chemistries (Continued)	Grade 1	Grade 2	Grade 3	Grade 4
Calcium (ionized)	3.0-3.4 mg/dL or 5.6-6.0 mg/dL	2.5-2.9 mg/dL or 6.1-6.5 mg/dL	2.0-2.4 mg/dL or 6.6-7.0 mg/dL	< 2 mg/dL or > 7.0 mg/dL
Calcium (corrected for albumin)	7.8- < 8.4 mg/dL or 10.6-11.5 mg/dL	7.0-7.7 mg/dL or 11.6-12.5 mg/dL	6.1-6.9 mg/dL or 12.6-13.5 mg/dL	< 6.1 mg/dL or > 13.5 mg/dL
Triglycerides	----------------------	400-750 mg/dL	751-1200 mg/dL	> 1200 mg/dL
Glucose	55-64 mg/dL or 116-160 mg/dL	40-54 mg/dL or 161-250 mg/dL	30-39 mg/dL or 251-500 mg/dL	< 30 mg/dL or > 500 mg/dL
Magnesium	1.0-< 1.2 mEq/L	0.9-< 1.0 mEq/L	N/A	N/A
**Phosphorus**	**2.0- < 2.2 mg/dL**	**1.5-1.9 mg/dL**	**1.0-1.4 mg/dL**	**< 1.0 mg/dL**
Bilirubin	1.3 - 1.8 mg/dL	1.9 - 3 mg/dL	3.1 - 6 mg/dL	> 6 mg/dL
Bicarbonate	16 - < 17 mEq/L	11 - 15.9 mEq/L	8 - 10.9 mEq/L	< 8 mEq/L
**Creatinine**	**An increase ≥ 0.5 mg/dL from Baseline**	**2.1-3.0 mg/dL**	**3.1-6.0 mg/dL**	**> 6.0 mg/dL**

Appendix 1: Gilead Sciences Modified NIAID Common Toxicity Grading Scale (Continued)

Serum Chemistries (Continued)	Grade 1	Grade 2	Grade 3	Grade 4
BUN	30 - 60 mg/dL	61 - 120 mg/dL	121 - 240 mg/dL	> 240 mg/dL
CK (unrelated to trauma, exercise, or IM injection)	M: 248 - 498 U/L<br>F: 211 - 423 U/L	M: 499 - 990 U/L<br>F: 424 - 845 U/L	M: 991 - 1980 U/L<br>F: 846 - 1690 U/L	M: > 1980 U/L<br>F: > 1690 U/L
For Patients With Normal LFTs at Baseline (< 2 x ULN)				
**AST**	M: 46 - 90 U/L<br>**F: 43 - 85 U/L**	M: 91 - 180 U/L<br>**F: 86 - 170 U/L**	M: 181 - 360 U/L<br>**F: 171 - 340 U/L**	M: > 360 U/L<br>**F: > 340 U/L**
**ALT**	M: 54 - 108 U/L<br>**F: 43 - 85 U/L**	M: 109- 215 U/L<br>**F: 86 - 170 U/L**	M: 216 - 430 U/L<br>**F: 171 - 340 U/L**	M: > 430 U/L<br>**F: > 340 U/L**

Appendix 1: Gilead Sciences Modified NIAID Common Toxicity Grading Scale (Continued)

For Patients With Normal LFTs at Baseline (< 2 x ULN) (Continued)	Grade 1	Grade 2	Grade 3	Grade 4
Alkaline Phosphatase				
Female: 15 - 59 y	138 - 275 U/L	276 - 550 U/L	551 -1100 U/L	>1100 U/L
>59 y	144- 288 U/L	289- 575 U/L	576- 1150 U/L	>1150 U/L
Male: 15 - 20 y	313 - 625 U/L	626 - 1250 U/L	1251 - 2500 U/L	>2500 U/L
21 - 59 y	138 - 275 U/L	276 - 550 U/L	551 - 1100 U/L	>1 100 U/L
> 59 y	144 - 288 U/L	289 - 575 U/L	576 - 1150 U/L	>1150 U/L
For Patients With Elevated LFTs at Baseline (> 2 x ULN)				
**AST**	M: 180- 252 U/L<br>**F: 170 - 238 U/L**	M: 253 - 360 U/L<br>**F: 239 - 340 U/L**	M: 361 - 540 U/L<br>**F: 341- 510 U/L**	M: > 540 U/L<br>**F: > 510 U/L**
**ALT**	M: 215 - 301 U/L<br>**F: 170 - 238 U/L**	M: 302- 430 U/L<br>**F: 239 - 340 U/L**	M: 431 - 645 U/L<br>**F: 341 - 510 U/L**	M: > 645 U/L<br>**F: > 510 U/L**

Appendix 1: Gilead Sciences Modified NIAID Common Toxicity Grading Scale (Continued)

For Patients With Elevated LFTs at Baseline (>_ 2 x ULN) (Continued)	Grade 1	Grade 2	Grade 3	Grade 4
Alkaline Phosphatase				
Female: 15- 59 y	550- 770 U/L	771- 1100 U/L	1101- 1650 U/L	> 1650 U/L
>59 y	575- 805 U/L	806- 1150 U/L	1151- 1725 U/L	>1725 U/L
Male: 15- 20 y	1250- 1750 U/L	1751- 2500 U/L	2501- 3750 U/L	>3750 U/L
20- 59 y	550- 770 U/L	771- 1100 U/L	1101- 1650 U/L	>1650 U/L
> 59 y	575- 805 U/L	806- 1150 U/L	1151- 1725 U/L	>1725 U/L
Amylase	97- 131 U/L	132- 175 U/L	176- 439 U/L	> 440
Lipase	1-60 yr:154 - 210 U/L<br>> 60 yr:198 - 270 U/L	1-60 yr:211 - 279 U/L<br>> 60 yr:271 - 360 U/L	1-60 yr:280 - 699 U/L<br>> 60 yr:361 - 900 U/L	1-60 yr: > 699<br>> 60 yr: > 900

Appendix 1: Gilead Sciences Modified NIAID Common Toxicity Grading Scale (Continued)

Urinalysis	Grade 1	Grade 2	Grade 3	Grade 4
Proteinuria	1+; 30-99 mg/dL	2+ to 3+; 100-1000 mg/dL	4+; > 1000 mg/dL	nephrotic syndrome
Glycosuria	1+	2+	3+	4+
Hematuria	Microscopic only, 1-10 RBCs	gross hematuria without clots or 10-100 RBCs	gross hematuria with clots or > 101 RBCs or too numerous to count	obstructive or Rx required
Respiratory				
Dyspnea	Dyspnea on significant exertion	dyspnea at normal level of activity	dyspnea at rest	requires ventilatory support
Cardiac Function	---------------------	Symptoms with ordinary Slight limitation of activity.	Symptoms with less than physical activity. Marked limitation of activity.	Symptoms with any activity or even at rest.
Hypertension	20-25% increase from baseline systolic	26-30% increase from	31-40% increase systolic	> 40% increase from systolic
Hypotension	20-25% decrease from baseline systolic	26-30% decrease from Systolic	31-40% decrease systolic	> 40% decrease from systolic

Appendix 1: Gilead Sciences Modified NIAID Common Toxicity Grading Scale (Continued)

Gastrointestinal	Grade 1	Grade 2	Grade 3	Grade 4
Oral discomfort/ Stomatitis	mild discomfort, no difficulty swallowing	difficulty swallowing but able to eat and drink fluids	unable to swallow solids	unable to drink fluids; requires IV fluids
Nausea	mild or transient, maintains reasonable food intake	moderate discomfort; some limitation of food intake	severe discomfort; minimal food intake for 3 or more days	life threatening, unable to ingest any food or fluid in 72 hours
Vomiting	mild or transient; 2-3 episodes in 24 hours OR 1-2 episodes per day lasting < 1 week	moderate or persistent; 4-5 episodes in 24 hours OR 1-2 episodes per day for > 1 week	severe; vomiting of all food/fluids in 24 hrs. OR orthostatic hypotension	life threatening hypotensive shock
Diarrhea	mild or transient, 3-4 loose stools/in 24 hours OR mild diarrhea lasting < 1 week	moderate or persistent, 5-7 loose stools in 24 hours OR 3-4 loose stools per day > 1 week	severe; bloody diarrhea or 8-9 loose stools in 24 hours OR orthostatic hypotension	life threatening; hypotensive shock OR hospitalization for IV fluids
Abdominal Pain	mild, occasional transient	moderate, transient, no or minimal Rx required	severe or requiring analgesic	severe with guarding peritoneal signs

Appendix 1: Gilead Sciences Modified NIAID Common Toxicity Grading Scale (Continued)

Neuro/Neuromuscular (Continued)	Grade 1	Grade 2	Grade 3	Grade 4
Neuro Cerebellar	Slight incoordination; dysdiadochokinesia	intention tremor; dysmetria; nystagmus	locomotor ataxia	incapacitated
Neuro-psych/Mood	Mild anxiety or mild depression	therapy required for moderate anxiety or moderate depression	needs assistance for severe anxiety or severe depression or severe mania	acute psychosis or incapacitated or hospitalization
Muscle strength	Subjective weakness; no objective symptoms	mild objective weakness; no decrease in function	objective weakness; function limited	paralysis
Headache	mild; no therapy required	moderate; non-narcotic analgesic therapy required	severe; responds to initial narcotic therapy	intractable; requiring repeated narcotic therapy
Peripheral Neuropathy	mild discomfort; no therapy required	moderate discomfort persisting for > 72 hours; non-narcotic analgesia required OR mild discomfort persisting for > 72 hours accompanied by loss of deep tendon reflex previously present	severe discomfort; marked antalgic gait; narcotic analgesia required with symptomatic improvement	incapacitating; intolerable discomfort; not improved OR unable to walk despite narcotic analgesia

Appendix 1: Gilead Sciences Modified NIAID Common Toxicity Grading Scale (Continued)

Neuro/Neuromuscular (Continued)	Grade 1	Grade 2	Grade 3	Grade 4
Myositis	minimal findings	Patients must have some (positive EMG or muscle biopsy) and one of the following:<br>1) mild to moderate muscle pain present > 4 weeks; may require nonsteroidal anti-inflammatory drugs<br>2) difficulty climbing stairs or rising from a sitting position but able to ambulate without assistance	Patients must have some myositis (positive EMG or muscle biopsy) and one of the following:<br>1) moderate to severe > 4 weeks requiring nonsteroidal anti-inflammatory drugs (NSAII)S)<br>2) needs some assistance with ambulation or general activities	Patients must have some myositis (positive EMG or muscle biopsy) and one of the following:<br>1) severe myalgias not exercise, requiring narcotics<br>2)muscle weakness inability to ambulate, requiring special care and assistance with mobilization<br>3) acute rhabdomyolysis muscle necrosis and edema, moderate to severe muscle weakness with inability to ambulate or mobilize without<br>4) acute rhabdomyolysis with electrolyte imbalance in renal failure

Appendix 1: Gilead Sciences Modified NIAID Common Toxicity Grading Scale (Continued)

Skin / Allergy	Grade 1	Grade 2	Grade 3	Grade 4
Allergic reaction	pruritus without rash	localized urticaria	generalized urticaria, angioedema	anaphylaxis
Local reaction	Erythema OR tenderness	induration < 10 mm or phlebitis or inflammation	induration > l0mm or ulceration	necrosis of skin
Cutaneous	Erythema OR pruritus	diffuse maculopapular rash, dry	vesiculation, moist ulceration	exfoliative dermatitis, membrane involvement, OR Stevens-Johnson OR erythema multiforme, OR necrosis requiring surgery
OTHER MEASUREMENTS				
Fever oral, (in absence infection) > 12 hrs.	37.7-38.5°C or 100.0-	38.6-39.5°C or 101.6-102.9°F	39.6-40.5°C or 103-1057	> 40.5°C or >
Fatigue	normal activity reduced < 25%	normal activity reduced 25-50%	normal activity reduced > 50%, cannot work	unable to care for self
Other toxicity not in table	Transient or mild discomfort; requiring no limitation of activity; no therapy	mild-moderate impact on activity; requiring some assistance and medical intervention	marked impact on activity; requiring some assistance and medical intervention	complete disability; requiring significant assistance and medical intervention and/or hospitalization

APPENDIX 2: MANAGEMENT OF CLINICAL AND LAB ADVERSE EVENTS

Other than creatinine elevation

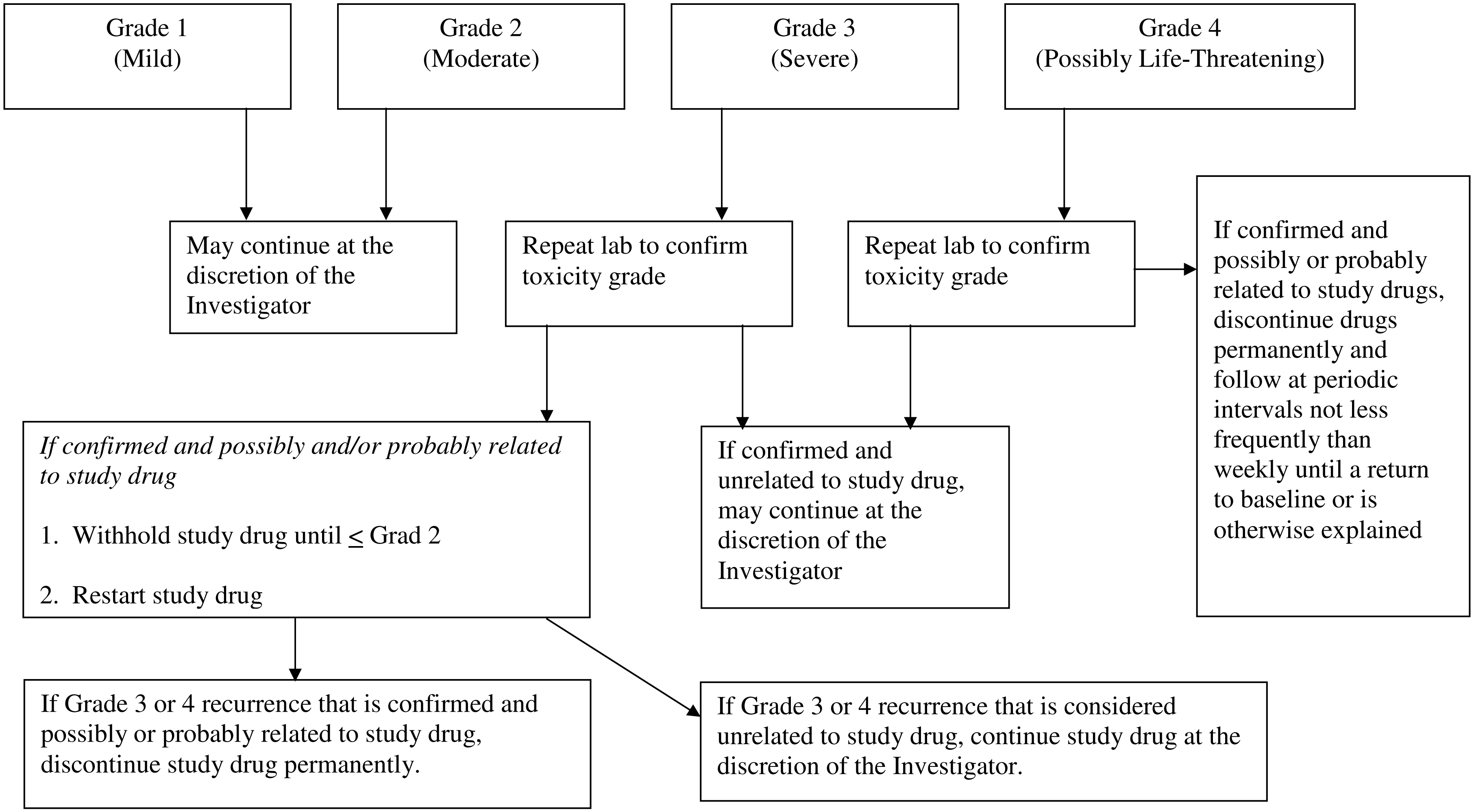

APPENDIX 3: MANAGEMENT OF CREATININE ELEVATIONS

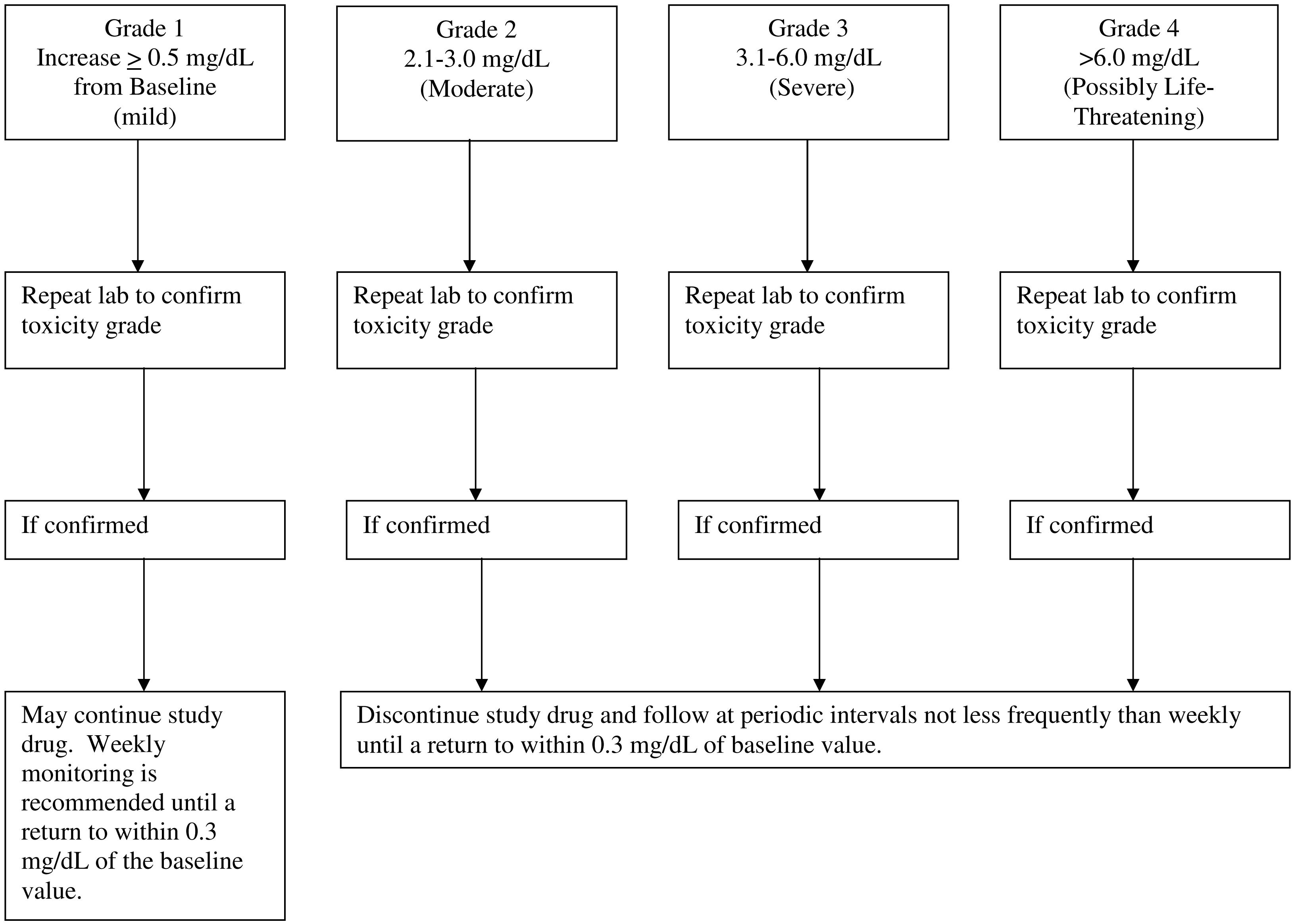

APPENDIX 4: STUDY ACTIVITIES CHART

Event	Screening	Enrollment	Follow-Up	Final Visit
**Informed consent**	◆	◆	◆	◆
**Eligibility criteria assessment**	◆	◆	◆	
**Assign Participant ID number**	◆			
**Pre-test HIV counseling**	◆	◆	◆	◆
**HIV test**	◆	◆	◆	◆
**Urine pregnancy test**	◆	◆	◆	◆
**Obtain contact information**	◆	◆	◆	
**Physical exam**	◆	(if indicated)	◆ (months 1, 3, 6, 9, 12)	◆
**Blood draw for labs**	◆		◆ (months 1, 3, 6, 9, 12)	◆
**Counseling for STI prevention**	◆	◆	◆	◆
**Interview**	◆	◆	◆	◆
**Post-test HIV counseling & test results**	◆	◆	◆	◆
**Randomization**		◆		
**Product supplies given**		◆	◆	
**AE assessment**			◆	◆
**Concomitant medications**	◆	◆	◆	◆
